# Scaffold Repurposing of In-House Small Molecule Candidates Leads to Discovery of First-in-Class CDK-1/HER-2 Dual Inhibitors: In Vitro and In Silico Screening

**DOI:** 10.3390/molecules26175324

**Published:** 2021-09-01

**Authors:** Ahmed Elkamhawy, Usama M. Ammar, Sora Paik, Magda H. Abdellattif, Mohamed H. Elsherbeny, Kyeong Lee, Eun Joo Roh

**Affiliations:** 1 College of Pharmacy, Dongguk University-Seoul, Goyang 10326, Korea; 2 Department of Pharmaceutical Organic Chemistry, Faculty of Pharmacy, Mansoura University, Mansoura 35516, Egypt; 3 Strathclyde Institute of Pharmacy and Biomedical Sciences, University of Strathclyde, 161 Cathedral Street, Glasgow G4 0NR, UK; u.ammar@outlook.com; 4 Chemical Kinomics Research Center, Korea Institute of Science and Technology (KIST), Seoul 02792, Korea; sora0960@naver.com (S.P.); Mohamed.alsherbeny@pharma.asu.edu.eg (M.H.E.); 5 Department of Chemistry, College of Science, Taif University, P.O. Box 11099, Taif 21944, Saudi Arabia; m.hasan@tu.edu.sa; 6 Division of Bio-Medical Science & Technology, KIST School, University of Science and Technology, Seoul 02792, Korea; 7 Pharmaceutical Chemistry Department, Faculty of Pharmacy, Ahram Canadian University, Giza 12566, Egypt

**Keywords:** CDK-1/cyclin B, HER-2, anti-proliferative, anti-cancer, molecular docking, drug repurposing

## Abstract

Recently, multitargeted drugs are considered a potential approach in treating cancer. In this study, twelve in-house indole-based derivatives were preliminary evaluated for their inhibitory activities over VEGFR-2, CDK-1/cyclin B and HER-2. Compound **15l** showed the most inhibitory activities among the tested derivatives over CDK-1/cyclin B and HER-2. Compound **15l** was tested for its selectivity in a small kinase panel. It showed dual selectivity for CDK-1/cyclin B and HER-2. Moreover, in vitro cytotoxicity assay was assessed for the selected series against nine NCI cell lines. Compound **15l** showed the most potent inhibitory activities among the tested compounds. A deep in silico molecular docking study was conducted for compound **15l** to identify the possible binding modes into CDK-1/cyclin B and HER-2. The docking results revealed that compound **15l** displayed interesting binding modes with the key amino acids in the binding sites of both kinases. In vitro and in silico studies demonstrate the indole-based derivative **15l** as a selective dual CDK-1 and HER-2 inhibitor. This emphasizes a new challenge in drug development strategies and signals a significant milestone for further structural and molecular optimization of these indole-based derivatives in order to achieve a drug-like property.

## 1. Introduction

Cancer is a multifactorial disease defined by a number of uncontrolled cellular growth and signaling pathways. CDK-1 (cyclin-dependent kinase type 1) and HER-2 (epidermal growth factor receptor type 2) are considered important key elements in human cells since they play fundamental roles in signal transduction and cellular growth [1,2].

**Figure 1:**
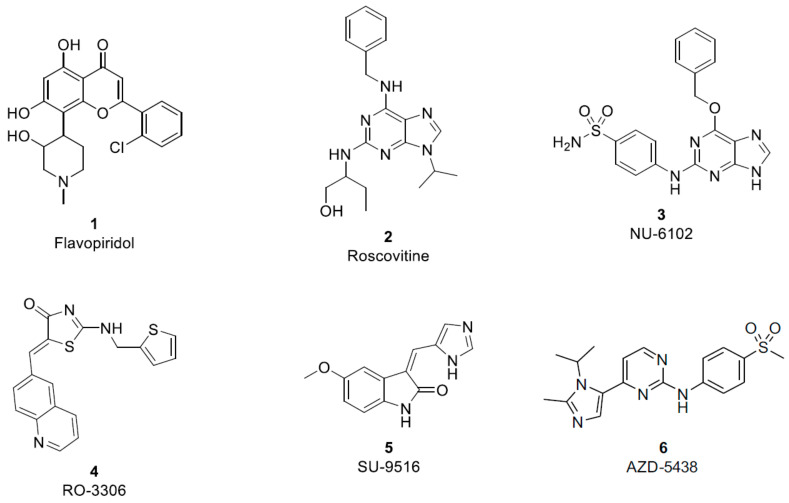
CDK-1/cyclin B inhibitors.

CDKs are among the protein kinase family which have a prominent and significant impact in cell cycle regulation [3]. They are serine–threonine kinases, which phosphorylate their substrate on serine and threonine amino acid residues [4]. CDK-1 (one member of the CDK family) builds an active form with cyclin B (CDK-1/cyclin B), which in turn lead to cell cycle progression at M phase [5,6]. Mutation and overexpression of this active complex (CDK-1/cyclin B) can lead to abnormal cell growth and proliferation [7,8]. In recent years, kinase inhibition has become a major area for therapeutic intervention to treat abnormal cell proliferation [9]. A number of CDK inhibitors (CDIs) have been developed (Figure 1). Among them, flavopiridol (**1**, Alvocidib^TM^, Sanofi) [10], a broad spectrum CDK inhibitor with activity against CDK-1, CDK-2 and CDK-4, is currently in clinical trial phase 2 as an anti-proliferative agent [11]. It was reported that CDIs compete with ATP (adenosine triphosphate) at its binding site in the CDK-1/cyclin B complex causing inhibition of phosphorylation and further suppression of other signaling components, leading finally to blocking of the other important transcriptional activators for M phase [12].

**Figure 2:**
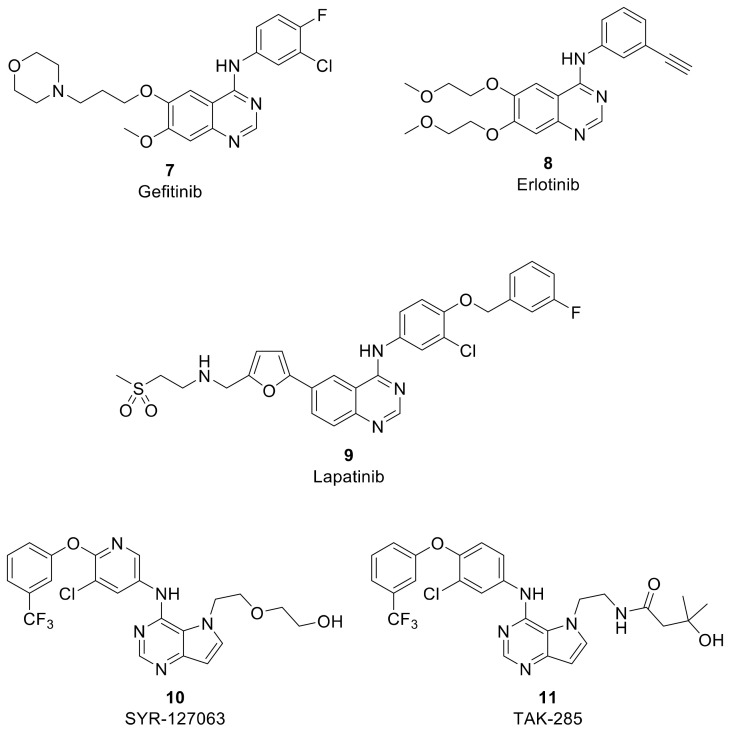
HER-2 TK inhibitors.

HER-2 (ErbB-2, epidermal growth factor receptor-2) is one member of the ErbB receptor family and has an essential role in cell proliferation, survival, migration, adhesion and differentiation [13,14]. Overexpression of HER-2 leads to uncontrolled activation of the receptor and hence to cell growth in tumors [15,16]. Therefore, targeting human epidermal growth factor-2 (HER-2) is a common approach for cancer therapy [17,18]. Tyrosine kinase domain (TK) of HER-2 has been identified as an efficient target in inhibiting and suppressing overexpressed HER-2 cancer cells [19,20]. A number of inhibitors have been reported and approved by the FDA (Food and Drug Administration) to inhibit the TK domain of HER-2 through binding to the adenosine triphosphate (ATP) binding site and blocking the key triggering phosphorylation step in the cell growth signaling cascade [21] (Figure 2). Gefitinib (**7**, Iressa^TM^, AstraZeneca) [22], erlotinib (**8**, Tarceva^TM^, Genentech) [23] and lapatinib (**9**, Tykerb^TM^, GlaxoSmithKline) [24,25] are examples of reported HER-2 TK inhibitors. However, acquired resistance has been developed for these drugs after long term treatment [24,26]. Therefore, multitarget anticancer therapies have emerged as a promising treatment strategy through simultaneous modulation of a network of tumor-related targets, to overcome both acquired drug resistance and drug toxicity [27,28].

Lately, drug repurposing has been gaining significance in addressing novel clinical uses of existing therapeutic agents [29,30,31]. In the present study, our research group applied the concept of drug repurposing to twelve in-house *N*-substituted indole-based analogues (reported as potent inhibitors of MAO-B enzyme; monoamine oxidase type B) [32]. A literature search revealed that indole-based derivatives displayed broad kinase inhibitory activities [33,34]. In vitro enzyme assays and cytotoxicity evaluation were carried out for selected candidates to investigate their kinase inhibitory activities. In addition, a deep in silico molecular docking was conducted to investigate the binding modes into the binding site of the selected proteins.

## 2. Results and Discussion

### 2.1. Chemistry

**Scheme 1:**
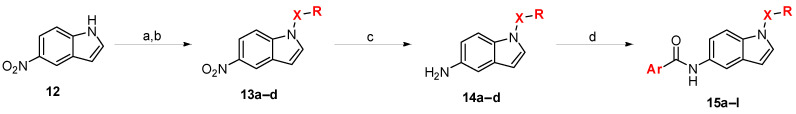
Reagents and conditions. **a**, dimethyl carbonate, K_2_CO_3_, DMF, reflux, 3 h (**13a**); **b**, appropriate aryl halide, NaH, DMF, 0–100 °C, 24 h (**13b–d**); **c**, Fe, NH_4_Cl, EtOH/H_2_O, reflux, 1 h; **d**, appropriate aryl carboxylic acid, HATU, DIPEA, DMF, mw, 116 °C, 45 min.

The scale-up synthesis of the tested compounds was designed and carried out as depicted in Scheme 1. 5-nitroindole (**12**) was treated with dimethyl carbonate (methyl donor) in the presence of potassium carbonate as basic catalyst to afford the *N*-methyl derivative (**13a**) [35], while compounds **13b–d** were synthesized by treating 5-nitroindole with appropriate aryl halide in presence of sodium hydride as a base [36,37]. Reduction of compounds **13a–d** was achieved by iron in acidic medium to afford amino-based derivatives (**14a–d**) [38]. Final target compounds (**15a–l**) (Table 1 and Supplementary Table S1) were synthesized by treating amino-based derivatives (**14a–d**) with appropriate heteroaryl carboxylic acid using HATU as coupling agent in presence of Hunig’s base (DIPEA).

### 2.2. Biological Evaluation

#### 2.2.1. In Vitro Kinase Inhibitory Assay

Preliminary biological screening was designed to test the kinase inhibitory activity of the selected library (**15a–l**) over three kinases (VEGFR-2; vascular endothelial growth factor receptor type 2, CDK-1/cyclin B and HER-2). The selected derivatives were tested in a single dose duplicate mode at a concentration of 10 μM. Control compound, staurosporine, was tested in 10-dose IC_50_ (half maximal inhibitory concentration) mode with 4-fold serial dilution starting at 20 μM. Reactions were carried out at 10 μM ATP.

The results show that the selected indole-based derivatives (**15a–k**) did not display any inhibitory activities over 3 kinases. However, Compound **15l** (pyridazine-bearing derivative with carbonyl linker and terminal F-substituted phenyl ring) exhibited potent inhibitory activities over both CDK-1/cyclin B and HER-2 (Act% = 51% and 52%, respectively) compared to that of the tested compounds **15a–k** (Act% > 83%) (Table 2).

**Figure 3:**
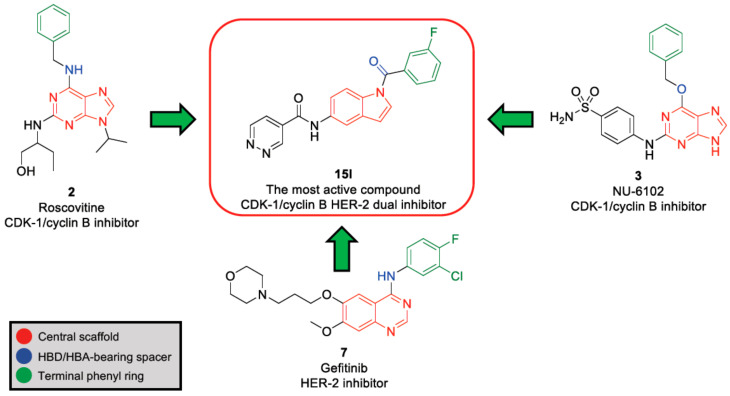
Structural correlation between the most active compound (**15l**) and CDK-1/cyclin B inhibitors (**2**, roscovitine and **3**, NU-6102) and HER-2 inhibitor (**7**, gefitinib).

The results suggest that the carbonyl-bearing linker along with the terminal substituted phenyl ring with F group may have a prominent role in binding into the active site of the kinase domain of CDK-1/cyclin B, as well as HER-2. This suggestion can be explained by the degree of similarity in the chemical group distribution around the main scaffold (Figure 3); HBD (hydrogen bond donor)/HBA (hydrogen bond acceptor)-bearing spacer in CDK-1/cyclin B inhibitors (**2**, roscovitine and **3**, NU-6102) and the HBD/HBA-bearing spacer along with terminal phenyl ring with halogen substitution in HER-2 inhibitors (**7**, gefitinib). These two factors (HBD/HBA-bearing spacer and terminal phenyl ring with halogen substitution) can participate in the dual inhibition of both CDK-1/cyclin B and HER-2 kinase domain.

To investigate the selectivity profile of the most active compound (**15l**), we treated this compound as in the previous protocol with a small kinase panel (aurora, BRAF V600A; V600A mutated rapidly accelerated fibrosarcoma kinase type B, HER-2, CDK-1/cyclin A, CDK-1/cyclin B and CDK-1/cyclin E) (Table 3). The results revealed that compound **15l** reserved its dual and selective inhibitory activities over CDK-1/cyclin B and HER-2. It did not show any inhibitory activities over the rest of the kinase panel.

#### 2.2.2. In Vitro Cytotoxicity Assay

All compounds under study (**15a–l**) were submitted to the National Cancer Institute (NCI) and selected for one-dose-testing over NCI human cancer cell lines (leukemia, non-small cell lung cancer, colon cancer, CNS (central nervous system) cancer, melanoma, ovarian cancer, renal cancer, prostate cancer and breast cancer). A few tested derivatives exhibited moderate activities over human renal cancer cell lines (Table 4 and Figure S1). Most of the tested compounds showed poor cytotoxic activities over NCI human cancer cell lines (Table S2 and Figures S2–S13). The results revealed that the A498 sub-panel renal cancer cell line was the most sensitive (G%^A498^ = 23%, **15d**; 39%, **15f** and 41%, **15h**).

Compound **15l** (the most active compound in the in vitro kinase inhibitory assay) showed moderate cytotoxic activity over renal cancer cell lines (G%^A498^ = 56%). The deprived cytotoxic activity of tested compounds can be referred to the poor cell permeability or drug efflux mechanism, washing the compound out rapidly. The relative sensitivity of the human renal cancer cell line can suggest a new correlation between the dual inhibition of CDK-1/cyclin B and HER-2 and their role in treating renal cancer.

### 2.3. In Silico Molecular Docking

A molecular docking experiment was conducted to predict the binding modes for the most active compound (**15l**) into the active site of the kinase domain of both CDK-1/cyclin B and HER-2 (PDB ID: 5LQF [39] and 3RCD [40], respectively). The docking experiment was carried out for the reference ligands of both kinase domains (NU-6102 (**3**) for CDK-1/cyclin B and TAK-285 (**11**) for HER-2) to validate the docking protocol. The validation step revealed that both reference ligands (**3** and **11**) exhibited identical binding modes, as well as binding forces with the key amino acid residues inside the binding site, for both CDK-1/cyclin B and HER-2, respectively, as reported in the protein data bank [39,40]. A virtual docking screening was conducted for compound **15l** in both kinase domains.

**Figure 4:**
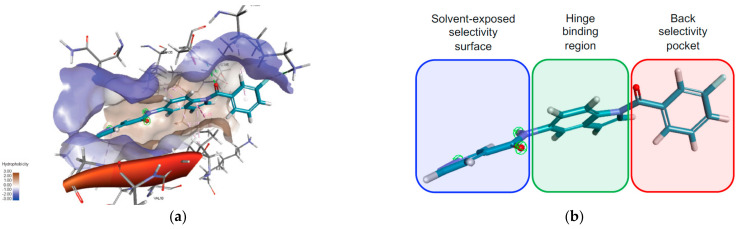
Binding mode of compound **15l** into CDK-1/cyclin B active site (PDB ID: 5LQF); (**a**): 3D visualization shows the orientation of compound **15l** binding motifs in the main regions of the active site; (**b**): graphical illustration of orientation of compound **15l** binding motifs in the CDK-1/cyclin B active site. Indole-based central motif occupied hinge binding region (green color), 3-fluorophenyl ring aligned into the back groove (red color) and terminal pyridazine ring travelled out towards solvent-exposed surface (blue color).

**Figure 5:**
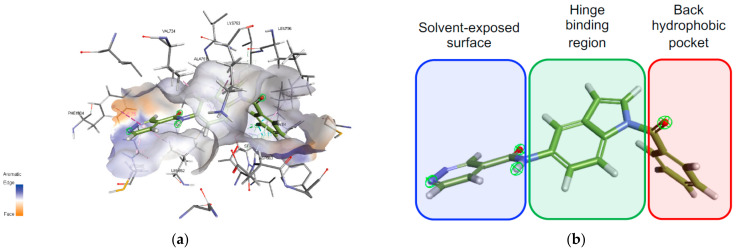
Binding mode of compound **15l** into HER-2 active site (PDB ID: 3RCD); (**a**): 3D visualization showed the orientation of compound **15l** binding motifs in the main regions of the active site; (**b**): graphical illustration of orientation of compound **15l** binding motifs in the HER-2 active site. Indole-based central motif occupied hinge binding region (green color), 3-fluorophenyl ring aligned into the back groove (red color) and terminal pyridazine ring travelled out towards solvent-exposed surface (blue color).

The results revealed that compound **15l** showed hypothetical binding modes with the key binding areas inside the active site (Figure 4 and Figure 5). In addition, it was seen that the main scaffold (indole-based scaffold) occupied the hinge region of both active sites with hydrophobic arene and H-bonding interactions. The right-handed aryl halide (3-fluorophenyl) moiety was directed into the back room of both kinase, while the left-handed heteroaryl ring (pyridazine ring) was revealed to the solvent-exposed surface.

It was suggested that the two linkers in compound **15l** (carbonyl and amide spacers that connect the central indole motif, the terminal aryl halide ring and terminal pyridazine ring, respectively) have a significant role in modulating the position of these pharmacophoric motifs in their corresponding grooves. For example, the carbonyl spacer will force the 3-fluorophenyl ring to be directed to the back pocket. In addition, it exhibited an additional hydrogen bond interaction with Asp 86 (aspartic acid) amino acid residue in CDK-1/cyclin B active site with a distance of 2.41 Å. Furthermore, the amide spacer (2-atoms-linker) gives a suitable distance between pyridazine and indole motif to stabilize the central scaffold in the hinge region, keeping the terminal pyridazine waving out to the solvent exposed area (Table 5).

## 3. Materials and Methods

### 3.1. Chemistry

The tested compounds were synthesized as reported in our previous publication [32]. The experimental method, along with spectral analysis for both key intermediates (**13a–d** and **14a–d**) and final compounds (**15a–l**), are cited in the Supplementary Materials. General methods for the chemical experiments were carried out as previously reported [41,42].

### 3.2. Biological Evaluation

#### 3.2.1. In Vitro Kinase Inhibitory Assay

The in vitro kinase inhibitory assay was conducted at Reaction Biology Corp. Kinase HotSpot service was used for screening the tested derivatives. The experiment protocol is reported in Reaction Biology Corp. website (www.reactionbiology.com, accessed on 10 March 2021) and described in detail in our previous reports [43,44].

#### 3.2.2. In Vitro Cytotoxicity Assay

The in vitro cytotoxic screening over nine NCI human cancer cell lines was conducted at the National Cancer Institute (NCI), Bethesda, MD, USA (dtp.cancer.gov), applying their standard protocol (dtp.cancer.gov/discovery_development/nci-60/methodology.htm, accessed on 5 April 2021), which was followed in earlier reports [45,46].

### 3.3. In Silico Molecular Docking

Molecular docking study was conducted using Discovery Studio Client 21.1.0.20298 (Biovia Corp) (www.3ds.com, accessed on 7 April 2021). The X-ray structure of kinase domain of both CDK-1/cyclin B and HER-2 was downloaded from protein data bank (www.rcsb.org, assessed on 7 April 2021) (PDB ID: 5LQF [39] and 3RCD [40], respectively). Non-essential chains as well as water molecules were removed keeping only one kinase domain with its corresponding reference ligand. The bonds and bond orders of the amino acid chain were checked and corrected. The terminal residues were checked and adjusted.

CHARMm (chemistry at Harvard macromolecular mechanics) forcefield was applied to the protein domain. Momany-Rone forcefield was selected for the partial charge. The binding site was defined using the coordinates of the reference ligand. CDOCKER algorithm (CHARMm-based molecular dynamic scheme) [47] was used in this study to generate the most stable conformers with the binding sites. Random ligand conformations were generated (10 conformers) from the initial ligand structure through dynamic target temperature (100 K) followed by random rotations. The generated conformers were refined by grid-based (GRID-1) simulated annealing.

The docking protocol was validated by running an initial docking experiment (pre-docking) for the reference ligands (**3**, NU-6102 for PDB ID: 5lQF and **11**, TAK-285 for PDB ID: 3RCD). The docking experiment of compound **15l** was conducted to generate the 10 most possible conformers with the binding site. The generated conformers were visualized to investigate the binding interaction between the tested compound (**15l**) and key amino acid residues in the active site of both kinase domains (CDK-1/cyclin B and HER-2).

## 4. Conclusions

A group of indole-based in-house derivatives (**15a–l**) was screened biologically over a small kinase panel. Compound **15l** (with terminal 3-fluorophenyl and pyridazine rings along with carbonyl and amide connected linkers to indole moiety, respectively) showed the most potent dual inhibitory activities among the tested compounds over CDK-1/cyclin B and HER-2. In vitro cytotoxic activity was evaluated to the selected compounds over NCI human cancer cell lines. Limited lipophilicity of the tested compounds may account for the poor cell induction, and hence leads to the low cytotoxic activities. However, the tested series showed moderate cytotoxic activities over human renal cancer cell lines (A498). An in silico molecular docking study was carried out to investigate the possible hypothetical binding modes of compound **15l** (the most active compound among the tested series) into both CDK-1/cyclin B and HER-2. Compound **15l** exhibited interesting binding mode in the kinase binding sites. The indole central motif occupied the hinge regions of both kinases. Terminal 3-fluorophenyl ring was directed back to the hydrophobic pocket. Pyridazine ring was pointed out to the solvent exposure area. In vitro and in silico studies afforded the indole-based derivatives as a selective dual CDK-1 and HER-2 inhibitor. This can be considered as a potential scaffold for further drug development strategies including both structural and molecular optimization.

## Figures and Tables

**Table 1:** Key structure of compounds **15a–l**.

Compound	X	R	Ar
**15a**	CH_2_	H	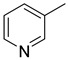
**15b**	CH_2_	H	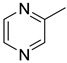
**15c**	CH_2_	H	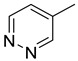
**15d**	CH_2_	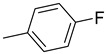	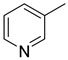
**15e**	CH_2_	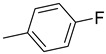	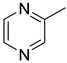
**15f**	CH_2_	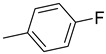	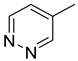
**15g**	CH_2_	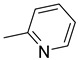	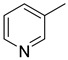
**15h**	CH_2_	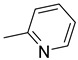	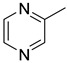
**15i**	CH_2_	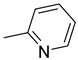	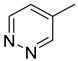
**15j**	C=O	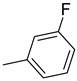	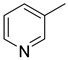
**15k**	C=O	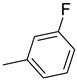	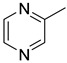
**15l**	C=O	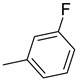	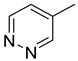

**Table 2:** Kinase activity (%) of the tested compounds (**15a–l**) over VEGFR-2, CDK-1/cyclin B and HER-2.

Compound	% Activity		
	VEGFR-2	CDK-1/cyclin B	HER-2
**15a**	96	91	95
**15b**	100	100	93
**15c**	100	91	99
**15d**	91	85	90
**15e**	98	88	86
**15f**	96	91	100
**15g**	100	92	83
**15h**	100	95	97
**15i**	85	89	87
**15j**	90	91	93
**15k**	90	92	92
**15l**	92	51	52

**Table 3:** Kinase activity (%) of 6 kinase panel with compound **15l**.

Kinase	% Activity
**Aurora**	86
**BRAF V600A**	100
**HER-2**	52
**CDK-1/cyclin A**	100
**CDK-1/cyclin B**	51
**CDK-1/cyclin E**	100
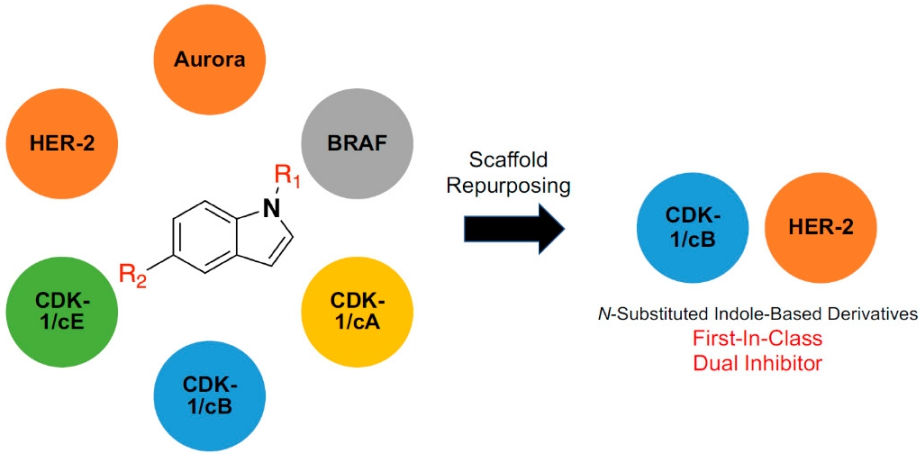	

**Table 4:** Growth % of NCI renal cancer cell lines treated with tested compounds (**15a–l**).

Compound	Growth%				
	Renal Cancer Cell Line				
	A498	CAKI-1	RXF 393	TK-10	UO-31
**15a**	76	96	100	98	97
**15b**	76	93	100	84	95
**15c**	68	69	65	73	65
**15d**	23	56	37	62	53
**15e**	53	48	57	53	54
**15f**	39	45	63	59	42
**15g**	55	70	53	72	61
**15h**	41	66	48	62	57
**15i**	57	66	59	60	59
**15j**	59	41	70	56	58
**15k**	87	71	68	65	72
**15l**	56	63	82	60	75

**Table 5:** Virtual binding interactions of compound **15l** into CDK-1/cyclin B and HER-2 kinase domain binding sites.

Kinase Domain	Compound 15l Binding Motifs		
	Pyridazine	Indole	3-Fluorophenyl
**CDK-1/cyclin B**	Val 18 Lys 33	Ile 10 Phe 83 Leu 135	Met 85 Lys 89
**HER-2**	Gly 804 Cyc 805 Leu 852 Phe 1004	Val 734 Ala 751 Lys 753 Leu 796	Ser 783 Leu 785 Asp 863

Val, valine; Lys, lysine; Ile, isoleucine; Phe, phenylalanine; Leu, leucine; Met, methionine; Gly, glycine; Cyc, cysteine; Ala, alanine; Ser, serine; Asp, Aspartic acid.

## Data Availability

Not applicable.

## Supplementary Materials

^1^HNMR, ^13^CNMR, purity and HRMS data of the compounds tested in this study, in addition to the detailed in vitro cytotoxicity data and NCI reports. Table S1: SMILES codes for the final target compounds **15a–l**, Table S2: Mean growth % of tested compounds **15a–l** over NCI human cancer cell lines, Figure S1: Graphical representation of in vitro cytotoxic evaluation of compounds **15a–l** over NCI human cancer cell lines. **Y-axis**, mean % inhibition; **X-axis**, target compounds. The graph shows that the renal cancer cell lines (deep blue) is more sensitive to the tested compounds compared to other NCI cancer cell lines, Figure S2: One dose mean growth % of compound **15a** over NCI human cancer cell lines, Figure S3: One dose mean growth % of compound **15b** over NCI human cancer cell lines, Figure S4: One dose mean growth % of compound **15c** over NCI human cancer cell lines, Figure S5: One dose mean growth % of compound **15d** over NCI human cancer cell lines, Figure S6: One dose mean growth % of compound **15e** over NCI human cancer cell lines, Figure S7: One dose mean growth % of compound **15f** over NCI human cancer cell lines, Figure S8: One dose mean growth % of compound **15g** over NCI human cancer cell lines, Figure S9: One dose mean growth % of compound **15h** over NCI human cancer cell lines, Figure S10: One dose mean growth % of compound **15i** over NCI human cancer cell lines, Figure S11: One dose mean growth % of compound **15j** over NCI human cancer cell lines, Figure S12: One dose mean growth % of compound **15k** over NCI human cancer cell lines, Figure S13: One dose mean growth % of compound **15l** over NCI human cancer cell lines.

## Sample Availability

Samples of the compounds **15a–l** are available from the authors.
