# Supplementary material for: Scaffold Repurposing of In-House Small Molecule Candidates Leads to Discovery of First-in-Class CDK-1/HER-2 Dual Inhibitors: In Vitro and In Silico Screening
Source: Molecules. 2021 Sep 1;26(17):5324. doi: 10.3390/molecules26175324 (PMC8433807; doi:10.3390/molecules26175324)
Supplement: Supplementary file 1 [file molecules-26-05324-s001.zip › molecules-1362206-SI.pdf]

Scaffold Repurposing of In-House Small Molecule Candidates Leads to Discovery of First-In-Class CDK-1/HER-2 Dual Inhibitors: *In vitro* and *In Silico* Screening

Ahmed Elkamhawy [1,2,*], Usama M. Ammar [3], Sora Paik [4], Magda H. Abdellattif [5], Mohamed H. Elsherbeny [4,6,7], Kyeong Lee [1,*] and Eun Joo Roh [4,6,*]

1 College of Pharmacy, Dongguk University-Seoul, Goyang 10326, Korea
2 Department of Pharmaceutical Organic Chemistry, Faculty of Pharmacy, Mansoura University, Mansoura 35516, Egypt
3 Strathclyde Institute of Pharmacy and Biomedical Sciences, University of Strathclyde, 161 Cathedral Street, Glasgow G4 0NR, Scotland, UK; u.ammar@outlook.com
4 Chemical Kinomics Research Center, Korea Institute of Science and Technology (KIST), Seoul 02792, Korea; sora0960@naver.com (S.P.); Mohamed.alsherbeny@pharma.asu.edu.eg (M.H.E.)
5 Department of Chemistry, College of Science, Taif University, P.O. Box 11099, Taif 21944, Saudi Arabia; m.hasan@tu.edu.sa
6 Division of Bio-Medical Science & Technology, KIST School, University of Science and Technology, Seoul 02792, Korea
7 Pharmaceutical Chemistry Department, Faculty of Pharmacy, Ahram Canadian University, Giza 12566, Egypt
* Correspondence: a_elkamhawy@mans.edu.eg or a.elkamhawy@dongguk.edu (A.E.); kaylee@dongguk.edu (K.L.); r8636@kist.re.kr (E.J.R.)

Supplementary Materials

Index	Page
S.1. Chemistry	2
S.2.1. *In vitro* cytotoxicity data	8
S.2.2. NCI reports	9

S.1. Chemistry

S.1.1. General

All solvents and reagents were obtained from commercial suppliers and used without further purification. Microwave-assisted synthetic procedure was applied in a Biotage Initiator+ apparatus operating in single mode; the microwave cavity producing controlled irradiation at 2.45 GHz (Biotage AB, Uppsala, Sweden). The reactions were carried out in sealed vessels by employing magnetic stirring with maintaining the desired temperature for the programmed time period. TLC was performed using glass sheets pre-coated with silica gel 60 F_{254} purchased by Merk. The NMR spectra were obtained on Bruker Avance 400. Column chromatography was carried out on Merck Silica Gel 60 (230–400 mesh). High-resolution spectra were performed on Waters ACQUITY UPLC BEH C18 1.7 μ–Q-TOF SYNAPT G2-Si High Definition Mass Spectrometry.

Table S1. SMILES codes for the final target compounds **15a–l**.

Compound	SMILES code
**15a**	C[N]1C=CC2=CC(=CC=C12)NC(=O)C3=CC=CN=C3
**15b**	C[N]1C=CC2=CC(=CC=C12)NC(=O)C3=NC=CN=C3
**15c**	C[N]1C=CC2=CC(=CC=C12)NC(=O)C3=CC=NN=C3
**15d**	FC1=CC=C(C[N]2C=CC3=CC(=CC=C23)NC(=O)C4=CC=CN=C4)C=C1
**15e**	FC1=CC=C(C[N]2C=CC3=CC(=CC=C23)NC(=O)C4=NC=CN=C4)C=C1
**15f**	FC1=CC=C(C[N]2C=CC3=CC(=CC=C23)NC(=O)C4=CC=NN=C4)C=C1
**15g**	O=C(NC1=CC=C2[N](CC3=NC=CC=C3)C=CC2=C1)C4=CC=CN=C4
**15h**	O=C(NC1=CC=C2[N](CC3=NC=CC=C3)C=CC2=C1)C4=NC=CN=C4
**15i**	O=C(NC1=CC=C2[N](CC3=NC=CC=C3)C=CC2=C1)C4=CC=NN=C4
**15j**	FC1=CC(=CC=C1)C(=O)[N]2C=CC3=CC(=CC=C23)NC(=O)C4=CC=CN=C4
**15k**	FC1=CC(=CC=C1)C(=O)[N]2C=CC3=CC(=CC=C23)NC(=O)C4=NC=CN=C4
**15l**	FC1=CC(=CC=C1)C(=O)[N]2C=CC3=CC(=CC=C23)NC(=O)C4=CC=NN=C4

S.1.2. Synthesis of 1-methyl-5-nitro-1*H*-indole (13a)

K_2CO_3 (55.3 mg, 0.24 mmol) was added to a solution of 5-nitroindole (**12**, 162.2 mg, 1.0 mmol) in DMF (5 mL). Dimethyl carbonate (0.17 mL, 2.0 mmol) was added and the reaction mixture was refluxed for 3 h, then cooled to 4–5 °C, followed by adding ice-cold water to the mixture. Precipitated solid was filtered and washed with water. Yellow solid, yield: 83%, mp: 164.7–165.9 °C, ^1H NMR (400 MHz, $CDCl_3$) δ 3.85 (s, 3H), 6.66 (d, *J* = 2.8 Hz, 1H), 7.21 (d, *J* = 2.9 Hz, 1H), 7.33 (d, *J* = 9.0 Hz, 1H), 8.11 (d, *J* = 9.0 Hz, 1H), 8.57 (s, 1H).

S.1.3. General procedure for synthesis of compounds 13b–d

NaH (60% in oil, 48.0 mg, 1.2 mmol) was added to a solution of 5-nitroindole (**12**, 162.2 mg, 1.0 mmol) dissolved in DMF (11 mL) at 0 °C. The reaction mixture was stirred at 0 °C for 10 min. The appropriate aryl halide (1.0 mmol) was added to the mixture. The mixture was then stirred at 100 °C for 24 h, cooled to room

temperature, and partitioned between EA and water using aqueous $NaHCO_3$. The organic layer was dried over anhydrous Na_2SO_4 and concentrated under reduced pressure. Finally, the residue was purified by column chromatography (SiO_2, EA/*n*-Hex) to get the target intermediate.

S.1.3.1. 1-(4-Fluorobenzyl)-5-nitro-1*H*-indole (13b)

Yellow solid, yield: 91%, mp: 126.4–127.0 °C, ^1H NMR (400 MHz, $CDCl_3$) δ 5.34 (s, 2H), 6.74 (s, 1H), 7.03 (d, *J* = 8.1 Hz, 2H), 7.08 (s, 2H), 7.28 (d, *J* = 10.6 Hz, 2H), 8.08 (d, *J* = 8.6 Hz, 1H), 8.60 (s, 1H).

S.1.3.2. 5-Nitro-1-(pyridin-2-ylmethyl)-1*H*-indole (13c)

Yellow solid, yield: 24%, ^1H NMR (400 MHz, $CDCl_3$) δ 5.49 (s, 2H), 6.76–6.80 (m, 2H), 7.20–7.23 (m, 1H), 7.33 (d, *J* = 9.0 Hz, 1H), 7.37 (d, *J* = 3.2 Hz, 1H), 7.59 (td, *J* = 1.6 Hz, 7.7 Hz, 1H), 8.08 (dd, *J* = 2.1 Hz, 9.1 Hz, 1H), 8.61 (d, *J* = 1.9 Hz, 2H).

S.1.3.3. (3-Fluorophenyl)(5-nitro-1*H*-indol-1-yl)methanone (13d)

Yellowish white solid, yield: 67%, mp: 151.0–152.0 °C, ^1H NMR (400 MHz, $CDCl_3$) δ 6.79 (d, *J* = 3.7 Hz, 1H), 7.35–7.40 (m, 1H), 7.47–7.50 (m, 2H), 7.54–7.60 (m, 2H), 8.25 (dd, *J* = 2.2 Hz, 9.1 Hz, 1H), 8.47–8.51 (m, 2H). ^{13}C NMR (100 MHz, $CDCl_3$) δ 109.24, 116.52, 116.53 ($J_{C–F}$ = 24.1 Hz), 117.20, 119.86 ($J_{C–F}$ = 21.1 Hz), 120.27, 125.06 ($J_{C–F}$ = 3.0 Hz), 130.06, 130.69 ($J_{C–F}$ = 5.0 Hz), 130.79, 135.29 ($J_{C–F}$ = 8.1 Hz), 138.97, 144.55, 162.51 ($J_{C–F}$ = 249.5 Hz), 167.08.

S.1.4. General procedure for synthesis of compounds 14a–d

Each of compounds **13a–d** (1.0 mmol) was dissolved in EtOH/H_2O (5/2), followed by adding NH_4Cl (267.5 mg, 5.0 mmol) and iron powder (558.5 mg, 10.0 mmol). The reaction mixture was refluxed for 1 h and filtered on celite then washed with EtOH. The residue was extracted using EA, water and aqueous $NaHCO_3$. The organic layer was dried over anhydrous Na_2SO_4 and concentrated under reduced pressure. The residue was purified by column chromatography (SiO_2, EA/*n*-Hex) to get the target intermediate.

S.1.4.1. 1-Methyl-1*H*-indol-5-amine (14a)

Brown solid, yield: 78%, mp: 103.0–103.9 °C, ^1H NMR (400 MHz, DMSO-d_6) δ 3.68 (s, 3H), 4.48 (s, 2H), 6.12 (d, *J* = 2.7 Hz, 1H), 6.56 (dd, *J* = 2.0 Hz, 8.6 Hz, 1H), 6.70 (d, *J* = 1.8 Hz, 1H), 7.09–7.12 (m, 2H).

S.1.4.2. 1-(4-Fluorobenzyl)-1*H*-indol-5-amine (14b)

Brown solid, yield: 80%, mp: 76.2–77.4 °C, ^{1}H NMR (400 MHz, DMSO-d_6) δ 4.49 (s, 2H), 5.27 (s, 2H), 6.18 (d, *J* = 2.8 Hz, 1H), 6.49 (dd, *J* = 1.8 Hz, 8.6 Hz, 1H), 6.69 (d, *J* = 1.6 Hz, 1H), 7.12 (t, *J* = 9.0 Hz, 3H), 7.20 (t, *J* = 8.5 Hz, 2H), 7.28 (d, *J* = 3.0 Hz, 1H). ^{13}C NMR (100 MHz, DMSO-d_6) δ 48.80, 99.98, 104.01, 110.64, 112.32, 115.65 ($J_{C\text{–}F}$ = 21.1 Hz), 128.94, 129.41 ($J_{C\text{–}F}$ = 8.0 Hz), 129.80, 130.07, 135.35 ($J_{C\text{–}F}$ = 3.0 Hz), 142.00, 161.79 ($J_{C\text{–}F}$ = 243.5 Hz).

S.1.4.3. 1-(Pyridin-2-ylmethyl)-1*H*-indol-5-amine (14c)

Light orange solid, yield: 72%, mp: 94.1–95.3 °C, ^{1}H NMR (400 MHz, DMSO-d_6) δ 4.51 (s, 2H), 5.38 (s, 2H), 6.22 (s, 1H), 6.49 (d, *J* = 8.2 Hz, 1H), 6.71 (s, 1H), 6.86 (d, *J* = 7.7 Hz, 1H), 7.07 (d, *J* = 8.5 Hz, 1H), 7.26–7.30 (m, 2H), 7.69 (t, *J* = 6.8 Hz, 1H), 8.54 (d, *J* = 3.5 Hz, 1H). ^{13}C NMR (100 MHz, DMSO-d_6) δ 51.69, 100.09, 104.01, 110.57, 112.34, 121.39, 122.91, 129.30, 129.76, 130.26, 137.45, 142.05, 149.55, 158.41.

S.1.4.4. (5-Amino-1*H*-indol-1-yl)(3-fluorophenyl)methanone (14d)

Orange solid, yield: 91%, mp: 91.7–92.9 °C, ^{1}H NMR (400 MHz, DMSO-d_6) δ 5.06 (s, 2H), 6.54 (d, *J* = 3.7 Hz, 1H), 6.68 (dd, *J* = 2.1 Hz, 8.7 Hz, 1H), 6.77 (d, *J* = 2.0 Hz, 1H), 7.19 (d, *J* = 3.7 Hz, 1H), 7.49–7.66 (m, 4H), 8.01 (d, *J* = 8.7 Hz, 1H). ^{13}C NMR (100 MHz, DMSO-d_6) δ 104.60, 109.35, 113.38, 116.07 ($J_{C\text{–}F}$ = 23.1 Hz), 116.81, 118.88 ($J_{C\text{–}F}$ = 21.1 Hz), 125.23 ($J_{C\text{–}F}$ = 3.0 Hz), 128.06, 128.23, 131.36 ($J_{C\text{–}F}$ = 8.1 Hz), 132.34, 137.14 ($J_{C\text{–}F}$ = 7.0 Hz), 146.25, 162.17 ($J_{C\text{–}F}$ = 245.5 Hz).

S.1.5. General procedure for synthesis of compounds 15a-l

To a MW vial, were successively added compounds **14a–d** (0.1 mmol), HATU (41.8 mg, 0.11 mmol), DIPEA (0.05 mL, 0.27 mmol), the appropriate heteroaryl carboxylic acid derivative (0.11 mmol) and DMF (12 mL) at room temperature. The MW vial was sealed and heated under MW conditions for 45 min at 116 °C. The reaction mixture was extracted using EA, water and brine. The organic layer was dried over anhydrous Na_2SO_4 and concentrated under reduced pressure. The residue was purified by column chromatography (SiO_2, EA/*n*-Hex) to get the desired target compound.

S.1.5.1. *N*-(1-Methyl-1*H*-indol-5-yl)nicotinamide (15a)

White solid, yield: 22%, mp: 164.2–164.9 °C, ^{1}H NMR (400 MHz, DMSO-d_6) δ 3.80 (s, 3H), 6.43 (d, *J* = 2.9 Hz, 1H), 7.34 (d, *J* = 3.0 Hz, 1H), 7.42–7.49 (m, 2H), 7.56–7.60 (m, 1H), 8.03 (d, *J* = 1.2 Hz, 1H), 8.32–8.34 (m, 1H),

8.77 (dd, *J* = 1.4 Hz, 4.8 Hz, 1H), 9.15 (d, *J* = 1.7 Hz, 1H), 10.33 (s, 1H). ^{13}C NMR (100 MHz, DMSO-d_6) δ 33.01, 100.84, 109.91, 112.89, 116.37, 123.92, 128.21, 130.74, 131.27, 131.41, 134.16, 135.77, 149.07, 152.27, 164.03.

S.1.5.2. *N*-(1-Methyl-1*H*-indol-5-yl)pyrazine-2-carboxamide (15b)

Brown solid, yield: 63%, mp: 172.5–174.0 °C, ^{1}H NMR (400 MHz, DMSO-d_6) δ 3.80 (s, 3H), 6.44 (s, 1H), 7.34 (s, 1H), 7.44 (d, *J* = 7.8 Hz, 1H), 7.61 (d, *J* = 7.1 Hz, 1H), 8.15 (s, 1H), 8.83 (s, 1H), 8.94 (s, 1H), 9.32 (s, 1H), 10.57 (s, 1H). ^{13}C NMR (100 MHz, DMSO-d_6) δ 33.01, 100.90, 110.00, 112.72, 116.22, 128.19, 130.67, 130.83, 134.26, 143.66, 144.34, 145.94, 147.90, 161.59.

S.1.5.3. *N*-(1-Methyl-1*H*-indol-5-yl)pyridazine-4-carboxamide (15c)

Yellowish green solid, yield: 28%, mp: 181.1–182.0 °C, ^{1}H NMR (400 MHz, DMSO-d_6) δ 3.81 (s, 3H), 6.45 (d, *J* = 3.0 Hz, 1H), 7.36 (d, *J* = 3.0 Hz, 1H), 7.47 (s, 2H), 8.04 (s, 1H), 8.15 (dd, *J* = 2.3 Hz, 5.3 Hz, 1H), 9.50 (dd, *J* = 1.0 Hz, 5.3 Hz, 1H), 9.69 (t, *J* = 1.0 Hz, 1H), 10.61 (s, 1H). ^{13}C NMR (100 MHz, DMSO-d_6) δ 33.03, 100.94, 110.08, 112.94, 116.15, 124.91, 128.20, 130.77, 130.94, 133.03, 134.35, 149.49, 152.53, 162.25.

S.1.5.4. *N*-(1-(4-Fluorobenzyl)-1*H*-indol-5-yl)nicotinamide (15d)

White solid, yield: 53%, mp: 200.0–200.4 °C, ^{1}H NMR (400 MHz, DMSO-d_6) δ 5.42 (s, 2H), 6.50 (d, *J* = 3.0 Hz, 1H), 7.15 (t, *J* = 8.9 Hz, 2H), 7.25–7.28 (m, 2H), 7.44 (dd, *J* = 8.8 Hz, 17.8 Hz, 2H), 7.53 (d, *J* = 3.0 Hz, 1H), 7.56–7.59 (m, 1H), 8.04 (s, 1H), 8.31 (d, *J* = 8.0 Hz, 1H), 8.76 (d, *J* = 3.5 Hz, 1H), 9.13 (d, *J* = 1.4 Hz, 1H), 10.34 (s, 1H). ^{13}C NMR (100 MHz, DMSO-d_6) δ 48.87, 101.72, 110.42, 113.08, 115.77 ($J_{C\text{–}F}$ = 22.1 Hz), 116.61, 123.93, 128.58, 129.51 ($J_{C\text{–}F}$ = 9.1 Hz), 130.20, 131.37, 131.49, 133.39, 134.98 ($J_{C\text{–}F}$ = 3.0 Hz), 135.78, 161.88 ($J_{C\text{–}F}$ = 242.5 Hz), 164.06.

S.1.5.5. *N*-(1-(4-Fluorobenzyl)-1*H*-indol-5-yl)pyrazine-2-carboxamide (15e)

Light yellow solid, yield: 73%, mp: 161.5–162.0 °C, HPLC purity: 6.02 min, 100%, ^{1}H NMR (400 MHz, DMSO-d_6) δ 5.42 (s, 2H), 6.51 (d, *J* = 3.0 Hz, 1H), 7.15 (t, *J* = 8.9 Hz, 2H), 7.26–7.30 (m, 2H), 7.46 (d, *J* = 8.8 Hz, 1H), 7.53–7.56 (m, 2H), 8.16 (d, *J* = 1.5 Hz, 1H), 8.82 (t, *J* = 1.5 Hz, 1H), 8.93 (d, *J* = 2.4 Hz, 1H), 9.32 (d, *J* = 1.2 Hz, 1H), 10.58 (s, 1H). ^{13}C NMR (100 MHz, DMSO-d_6) δ 48.88, 101.76, 110.48, 113.00, 115.78 ($J_{C\text{–}F}$ = 21.1 Hz), 116.53, 128.55, 129.55 ($J_{C\text{–}F}$ = 9.1 Hz), 130.27, 130.87, 133.48, 134.95 ($J_{C\text{–}F}$ = 4.0 Hz), 143.65, 144.35, 145.86, 147.92, 161.60, 161.88 ($J_{C\text{–}F}$ = 243.5 Hz). HRMS (ESI) *m/z* calcd. for $C_{20}H_{15}FN_4O$ $[M+H]^+$: 347.1308. Found: 347.1306.

S.1.5.6. *N*-(1-(4-Fluorobenzyl)-1*H*-indol-5-yl)pyridazine-4-carboxamide (15f)

Yellowish green solid, yield: 46%, mp: 212.6–213.1 °C, ^{1}H NMR (400 MHz, DMSO-d_6) δ 5.42 (s, 2H), 6.52 (d, *J* = 2.6 Hz, 1H), 7.15 (t, *J* = 8.8 Hz, 2H), 7.26 (t, *J* = 8.0 Hz, 2H), 7.41 (d, *J* = 8.0 Hz, 1H), 7.48 (d, *J* = 8.6 Hz, 1H), 7.54 (d, *J* = 2.8 Hz, 1H), 8.04 (s, 1H), 8.13 (d, *J* = 2.9 Hz, 1H), 9.48 (d, *J* = 4.7 Hz, 1H), 9.67 (s, 1H), 10.59 (s, 1H). ^{13}C NMR (100 MHz, DMSO-d_6) δ 48.89, 101.82, 110.57, 113.13, 115.80 ($J_{C–F}$ = 21.1 Hz), 116.40, 124.92, 128.58, 129.53 ($J_{C–F}$ = 8.1 Hz), 130.39, 130.99, 132.98, 133.59, 134.93 ($J_{C–F}$ = 3.0 Hz), 149.47, 152.54, 161.89 ($J_{C–F}$ = 243.5 Hz), 162.29.

S.1.5.7. *N*-(1-(Pyridin-2-ylmethyl)-1*H*-indol-5-yl)nicotinamide (15g)

Light brown solid, yield: 38%, mp: 142.9–144.3 °C, ^{1}H NMR (400 MHz, DMSO-d_6) δ 5.52 (s, 2H), 6.52 (d, *J* = 2.9 Hz, 1H), 6.95 (d, *J* = 7.8 Hz, 1H), 7.29 (t, *J* = 4.9 Hz, 1H), 7.41 (s, 2H), 7.52 (d, *J* = 2.9 Hz, 1H), 7.56–7.59 (m, 1H), 7.70–7.74 (m, 1H), 8.05 (s, 1H), 8.31 (d, *J* = 7.8 Hz, 1H), 8.55 (d, *J* = 4.2 Hz, 1H), 8.76 (d, *J* = 3.5 Hz, 1H), 9.13 (s, 1H), 10.32 (s, 1H). ^{13}C NMR (100 MHz, DMSO-d_6) δ 51.71, 101.76, 110.41, 113.10, 116.65, 121.51, 123.08, 123.93, 128.55, 130.59, 131.37, 131.52, 133.64, 135.79, 137.58, 149.07, 149.70, 152.31, 157.96, 164.08.

S.1.5.8. *N*-(1-(Pyridin-2-ylmethyl)-1*H*-indol-5-yl)pyrazine-2-carboxamide (15h)

Light yellow solid, yield: 39%, mp: 152.0–152.9 °C, ^{1}H NMR (400 MHz, DMSO-d_6) δ 5.51 (s, 2H), 6.52 (d, *J* = 3.0 Hz, 1H), 6.96 (d, *J* = 7.8 Hz, 1H), 7.26–7.29 (m, 1H), 7.41 (d, *J* = 8.8 Hz, 1H), 7.51–7.53 (m, 2H), 7.71 (td, *J* = 1.6 Hz, 7.6 Hz, 1H), 8.15 (d, *J* = 1.5 Hz, 1H), 8.54 (d, *J* = 4.6 Hz, 1H), 8.81 (d, *J* = 1.5 Hz, 1H), 8.92 (d, *J* = 2.4 Hz, 1H), 9.31 (s, 1H), 10.57 (s, 1H). ^{13}C NMR (100 MHz, DMSO-d_6) δ 51.70, 101.80, 110.47, 113.00, 116.56, 121.55, 123.09, 128.52, 130.67, 130.89, 133.74, 137.59, 143.66, 144.36, 145.87, 147.93, 149.71, 157.93, 161.62.

S.1.5.9. *N*-(1-(pyridin-2-ylmethyl)-1*H*-indol-5-yl)pyridazine-4-carboxamide (15i)

Yellow solid, yield: 85%, mp: 141.8–143.0 °C, ^{1}H NMR (400 MHz, DMSO-d_6) δ 5.52 (s, 2H), 6.54 (d, *J* = 2.7 Hz, 1H), 6.96 (d, *J* = 7.8 Hz, 1H), 7.28 (t, *J* = 5.3 Hz, 1H), 7.39–7.45 (m, 2H), 7.53 (d, *J* = 2.9 Hz, 1H), 7.71 (t, *J* = 6.9 Hz, 1H), 8.06 (s, 1H), 8.13–8.14 (m, 1H), 8.55 (d, *J* = 4.4 Hz, 1H), 9.49 (d, *J* = 5.1 Hz, 1H), 9.67 (s, 1H), 10.61 (s, 1H). ^{13}C NMR (100 MHz, DMSO-d_6) δ 51.70, 101.85, 110.57, 113.13, 116.44, 121.53, 123.10, 124.93, 128.55, 130.77, 131.01, 132.98, 133.84, 137.59, 149.48, 149.72, 152.55, 157.89, 162.29.

S.1.5.10. *N*-(1-(3-Fluorobenzoyl)-1*H*-indol-5-yl)nicotinamide (15j)

White solid, yield: 57%, mp: 212.9–214.2 °C, ^{1}H NMR (400 MHz, DMSO-d_6) δ 6.82 (d, *J* = 3.6 Hz, 1H), 7.43 (d, *J* = 3.6 Hz, 1H), 7.56–7.73 (m, 6H), 8.23 (d, *J* = 1.5 Hz, 1H), 8.30 (d, *J* = 8.9 Hz, 1H), 8.35 (d, *J* = 7.9 Hz, 1H),

8.78–8.79 (m, 1H), 9.16 (d, *J* = 1.5 Hz, 1H), 10.58 (s, 1H). ^{13}C NMR (100 MHz, DMSO-d_6) δ 109.57, 113.07, 116.36 ($J_{C–F}$ = 24.1 Hz), 116.38, 118.63, 119.34 ($J_{C–F}$ = 21.1 Hz), 123.99, 125.51, 129.29, 131.12, 131.37, 131.49 ($J_{C–F}$ = 8.1 Hz), 132.46, 135.64, 135.92, 136.63 ($J_{C–F}$ = 7.0 Hz), 149.15, 152.55, 162.20 ($J_{C–F}$ = 245.5 Hz), 164.47, 167.15.

S.1.5.11. *N*-(1-(3-Fluorobenzoyl)-1*H*-indol-5-yl)pyrazine-2-carboxamide (15k)

White solid, yield: 94%, mp: 221.3–222.2 °C, HPLC purity: 6.00 min, 100%, ^{1}H NMR (400 MHz, DMSO-d_6) δ 6.81 (d, *J* = 2.8 Hz, 1H), 7.43 (d, *J* = 3.0 Hz, 1H), 7.56 (t, *J* = 8.4 Hz, 1H), 7.61–7.67 (m, 3H), 7.86 (d, *J* = 8.9 Hz, 1H), 8.28 (d, *J* = 8.8 Hz, 1H), 8.32 (s, 1H), 8.85 (s, 1H), 8.96 (s, 1H), 9.34 (s, 1H), 10.85 (s, 1H). ^{13}C NMR (100 MHz, DMSO-d_6) δ 109.54, 113.16, 116.32, 116.37 ($J_{C–F}$ = 23.1 Hz), 118.77, 119.36 ($J_{C–F}$ = 20.1 Hz), 125.55, 129.33, 131.33, 131.49 ($J_{C–F}$ = 8.1 Hz), 132.61, 134.97, 136.59 ($J_{C–F}$ = 8.1 Hz), 143.72, 144.49, 145.65, 148.13, 162.11, 162.20 ($J_{C–F}$ = 245.5 Hz), 167.13. HRMS (ESI) *m*/*z* calcd. for $C_{20}H_{13}FN_4O_2$ [M+H]$^+$: 361.1101. Found: 361.1095.

S.1.5.12. *N*-(1-(3-Fluorobenzoyl)-1*H*-indol-5-yl)pyridazine-4-carboxamide (15l)

Yellowish white solid, yield: 75%, mp: 196.7–196.9 °C, ^{1}H NMR (400 MHz, DMSO-d_6) δ 6.84 (d, *J* = 3.6 Hz, 1H), 7.44 (d, *J* = 3.6 Hz, 1H), 7.54–7.71 (m, 5H), 8.16–8.18 (m, 1H), 8.23 (s, 1H), 8.33 (d, *J* = 8.9 Hz, 1H), 9.52 (d, *J* = 4.9 Hz, 1H), 9.70 (s, 1H), 10.85 (s, 1H). ^{13}C NMR (100 MHz, DMSO-d_6) δ 109.54, 113.17, 116.26, 116.51, 118.57, 119.37 ($J_{C–F}$ = 20.1 Hz), 125.01, 125.55 ($J_{C–F}$ = 3.0 Hz), 129.45, 131.40, 131.45, 131.53, 132.75 ($J_{C–F}$ = 5.0 Hz), 135.08, 136.57 ($J_{C–F}$ = 7.04 Hz), 149.46, 152.58, 162.20 ($J_{C–F}$ = 245.5 Hz), 162.75, 167.18.

S.2. Biological evaluation

S.2.1. In vitro cytotoxicity data

Table S2. Mean growth % of tested compounds **15a–l** over NCI human cancer cell lines.

Compound	Leukemia	NSCL*	Colon	CNS**	Melanoma	Ovarian	Renal	Prostate	Breast
**15a**	99.4	99.0	99.5	97.1	96.2	99.9	93.1	96.7	98.4
**15b**	94.9	98.6	99.5	97.7	95.8	99.8	91.1	99.3	97.3
**15c**	100.0	90.1	100.0	90.8	99.1	95.0	76.9	98.0	94.9
**15d**	78.1	85.3	90.9	84.0	90.3	89.2	59.4	83.6	79.3
**15e**	88.1	88.2	96.6	86.6	91.3	91.8	64.0	91.8	88.6
**15f**	83.4	71.8	92.9	81.1	89.7	88.7	58.1	79.5	87.5
**15g**	98.6	91.3	100.0	91.7	99.2	94.8	72.3	95.1	98.0
**15h**	97.3	88.9	99.1	89.3	98.4	93.2	67.1	97.8	97.0
**15i**	98.8	88.9	98.9	88.4	98.1	91.9	69.3	90.3	91.7
**15j**	86.3	91.8	98.1	93.0	98.3	94.4	67.8	90.2	97.6
**15k**	98.4	93.4	100.0	89.6	98.9	96.6	79.3	95.0	96.7
**15l**	94.3	91.5	99.1	91.3	98.6	95.9	76.2	99.2	98.8

* NSCL, non-small cell lung
** CNS, central nervous system

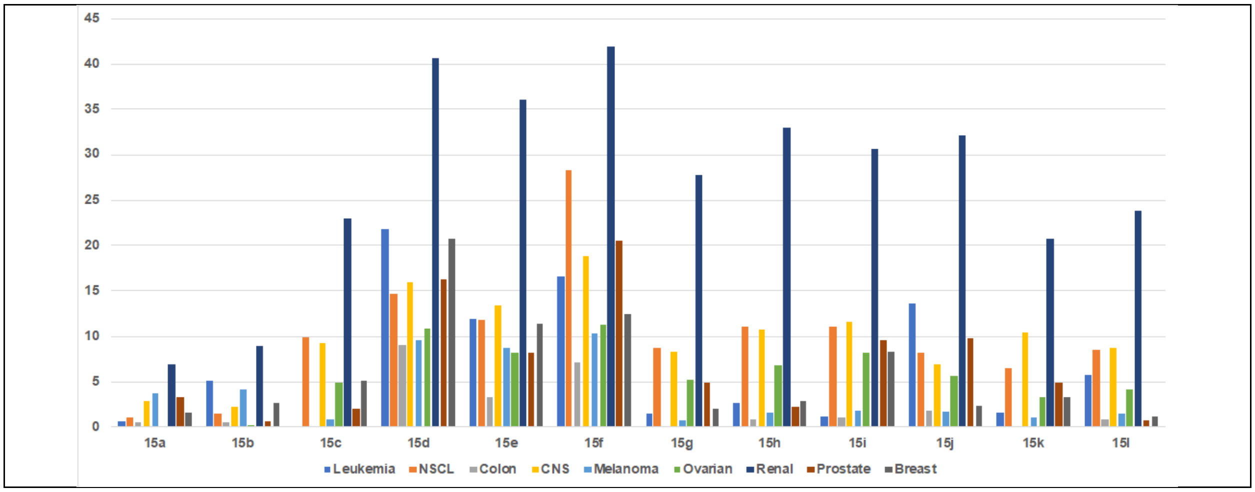

Figure S1. Graphical representation of *in vitro* cytotoxic evaluation of compounds **15a–l** over NCI human cancer cell lines. **Y-axis**, mean % inhibition; **X-axis**, target compounds. The graph shows that the renal cancer cell lines (deep blue) is more sensitive to the tested compounds compared to other NCI cancer cell lines.

S.2.2. NCI reports

Compound	NCI code	Compound	NCI code
**15a**	D-828867	**15g**	D-827532
**15b**	D-828866	**15h**	D-827531
**15c**	D-827533	**15i**	D-827530
**15d**	D-827526	**15j**	D-827529
**15e**	D-827525	**15k**	D-827528
**15f**	D-827524	**15l**	D-827527

Developmental Therapeutics Program	NSC : D-828867 / 1	Conc : 1.00E-5 Molar	Test Date : Mar 22, 2021
One Dose Mean Graph	Experiment ID : 2103OS00		Report Date : Apr 27, 2021

Panel/Cell Line	Growth Percent
**Leukemia**	
CCRF-CEM	99.56
HL-60(TB)	96.80
K-562	102.38
MOLT-4	101.97
RPMI-8226	106.96
SR	105.52
**Non-Small Cell Lung Cancer**	
A549/ATCC	102.44
EKVX	92.38
HOP-62	103.11
HOP-92	116.59
NCI-H226	100.09
NCI-H23	101.68
NCI-H322M	100.67
NCI-H460	102.62
NCI-H522	94.57
**Colon Cancer**	
COLO 205	109.73
HCC-2998	104.41
HCT-116	101.50
HCT-15	99.90
HT29	102.46
KM12	105.43
SW-620	96.68
**CNS Cancer**	
SF-268	103.01
SF-295	91.71
SF-539	101.98
SNB-19	99.00
SNB-75	91.65
U251	102.93
**Melanoma**	
LOX IMVI	105.99
MALME-3M	80.03
MDA-MB-435	93.15
SK-MEL-2	106.32
SK-MEL-28	103.40
SK-MEL-5	103.02
UACC-257	106.57
UACC-62	92.44
**Ovarian Cancer**	
IGROV1	106.54
OVCAR-3	110.52
OVCAR-4	99.01
OVCAR-5	104.55
OVCAR-8	102.96
NCI/ADR-RES	102.09
SK-OV-3	107.26
**Renal Cancer**	
786-0	81.32
A498	75.71
ACHN	101.57
CAKI-1	95.60
RXF 393	110.60
SN12C	96.71
TK-10	98.09
UO-31	97.39
**Prostate Cancer**	
PC-3	96.62
DU-145	96.85
**Breast Cancer**	
MCF7	96.69
MDA-MB-231/ATCC	104.21
HS 578T	108.87
BT-549	101.41
T-47D	99.99
MDA-MB-468	95.46
Mean	100.32
Delta	24.61
Range	40.88

Mean Growth Percent – Growth Percent

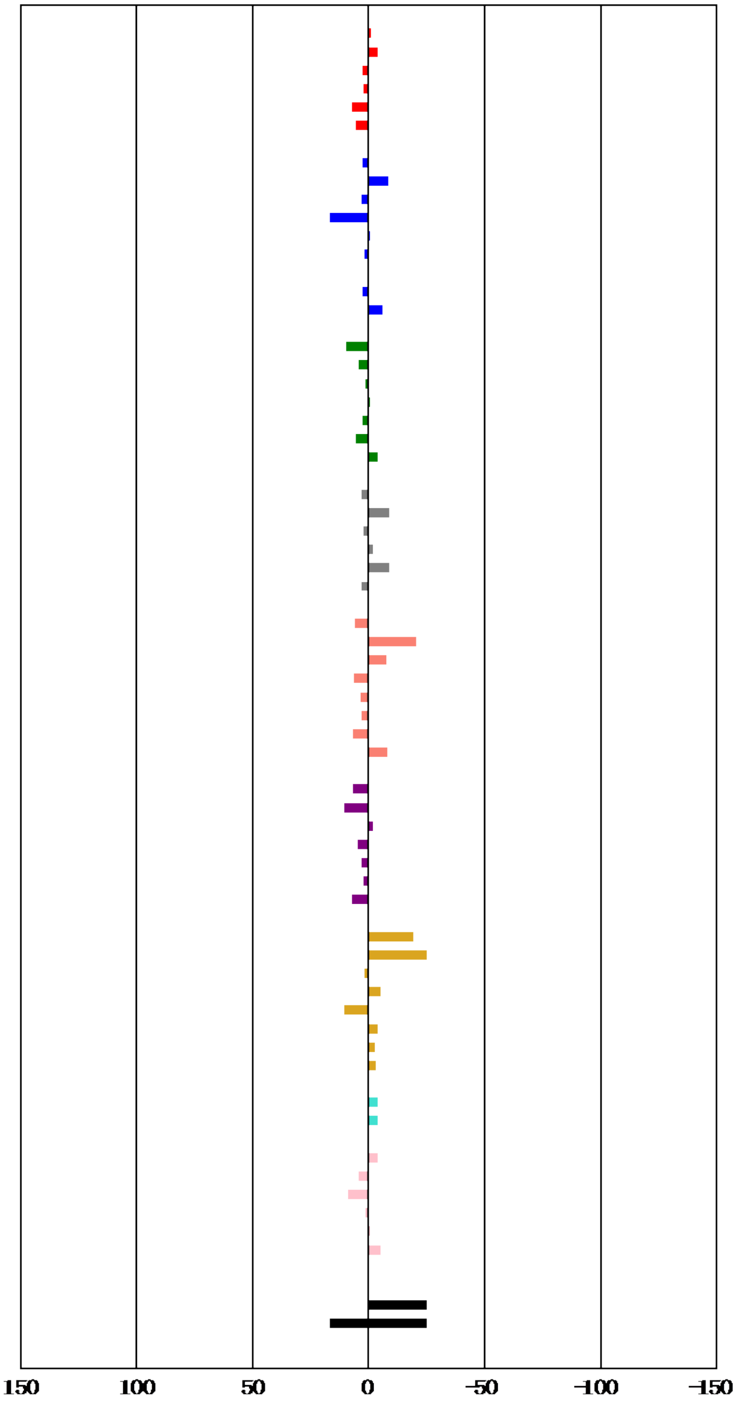

Figure S2. One dose mean growth % of compound **15a** over NCI human cancer cell lines.

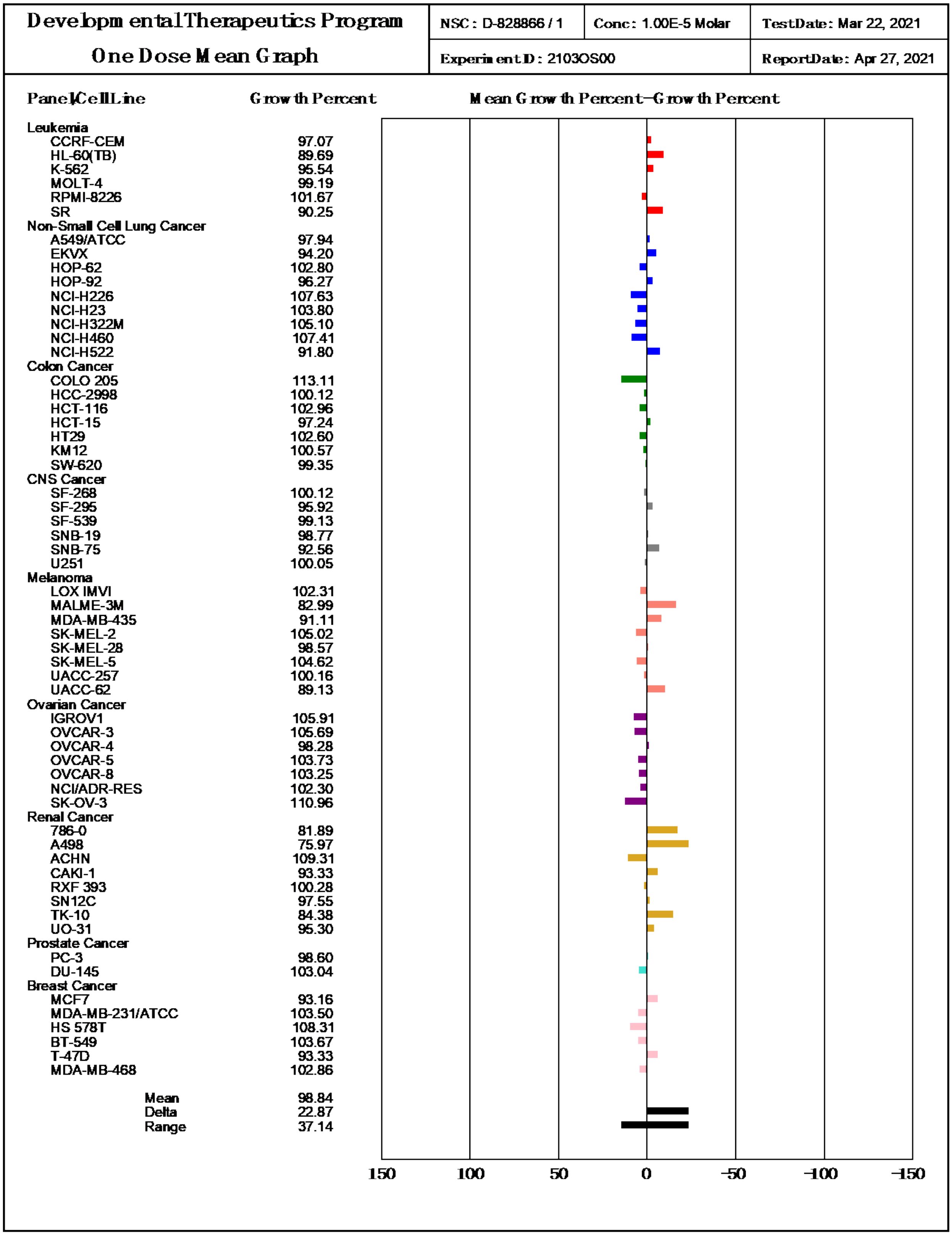

Figure S3. One dose mean growth % of compound **15b** over NCI human cancer cell lines.

Developmental Therapeutics Program	NSC : D-827533 / 1	Conc : 1.00E-5 Molar	Test Date : Jan 04, 2021
One Dose Mean Graph	Experiment ID : 2101OS70		Report Date : Mar 09, 2021

Panel/Cell Line	Growth Percent
**Leukemia**	
HL-60(TB)	123.00
K-562	111.53
MOLT-4	109.15
RPMI-8226	105.41
SR	107.63
**Non-Small Cell Lung Cancer**	
A549/ATCC	92.14
EKVX	66.94
HOP-62	75.72
HOP-92	103.68
NCI-H226	94.85
NCI-H23	90.95
NCI-H322M	106.92
NCI-H460	109.24
**Colon Cancer**	
COLO 205	113.25
HCC-2998	111.50
HCT-116	104.66
HCT-15	107.04
HT29	108.67
KM12	100.52
SW-620	102.94
**CNS Cancer**	
SF-268	87.51
SF-295	99.27
SF-539	99.45
SNB-19	100.46
SNB-75	58.53
U251	112.54
**Melanoma**	
LOX IMVI	103.57
MALME-3M	115.51
M14	106.68
MDA-MB-435	98.82
SK-MEL-2	97.99
SK-MEL-28	107.18
SK-MEL-5	103.84
UACC-257	103.61
UACC-62	95.27
**Ovarian Cancer**	
IGROV1	86.61
OVCAR-3	119.63
OVCAR-4	82.73
OVCAR-5	95.46
OVCAR-8	110.50
NCI/ADR-RES	107.66
SK-OV-3	110.30
**Renal Cancer**	
786-0	87.53
A498	67.75
ACHN	99.21
CAKI-1	69.27
RXF 393	64.90
SN12C	87.73
TK-10	73.39
UO-31	65.19
**Prostate Cancer**	
PC-3	101.80
DU-145	96.08
**Breast Cancer**	
MCF7	88.73
MDA-MB-231/ATCC	85.76
HS 578T	105.06
T-47D	106.59
MDA-MB-468	113.66
Mean	97.57
Delta	39.04
Range	64.47

Mean Growth Percent – Growth Percent

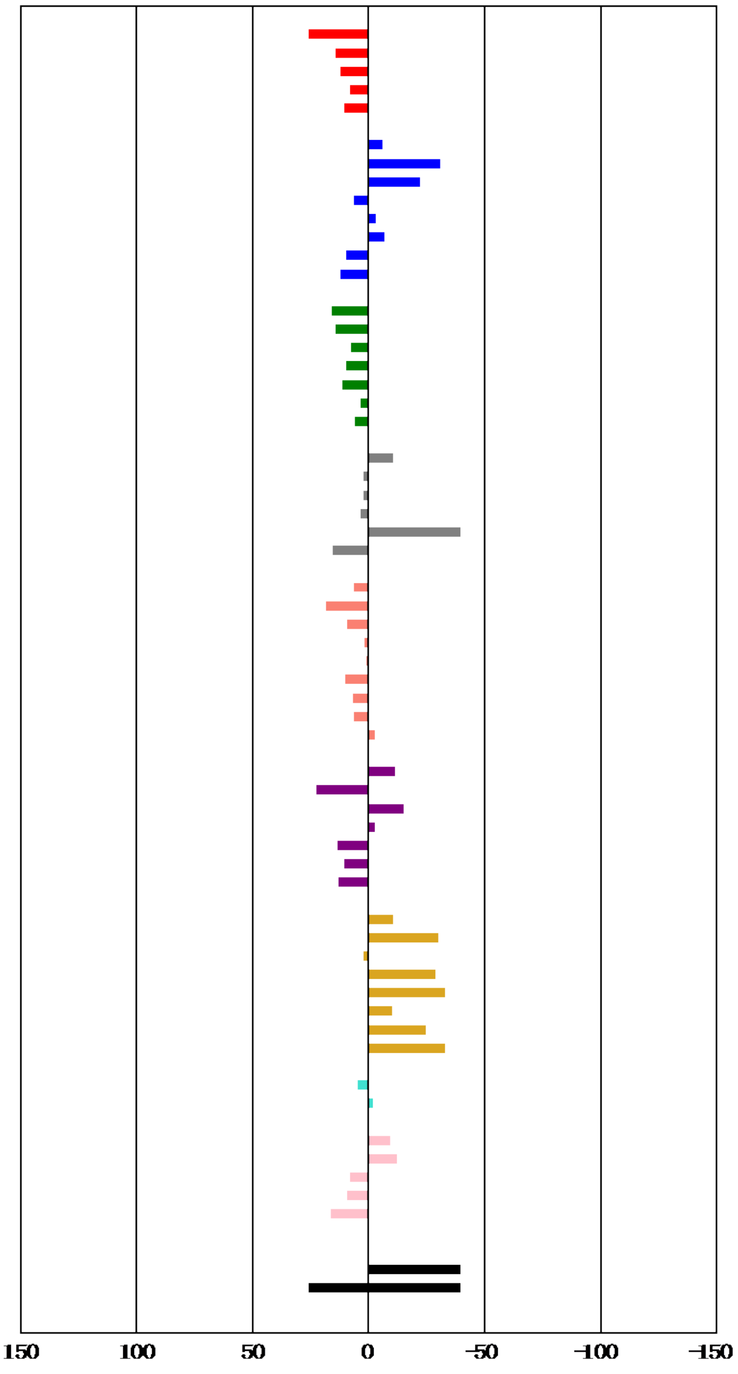

Figure S4. One dose mean growth % of compound **15c** over NCI human cancer cell lines.

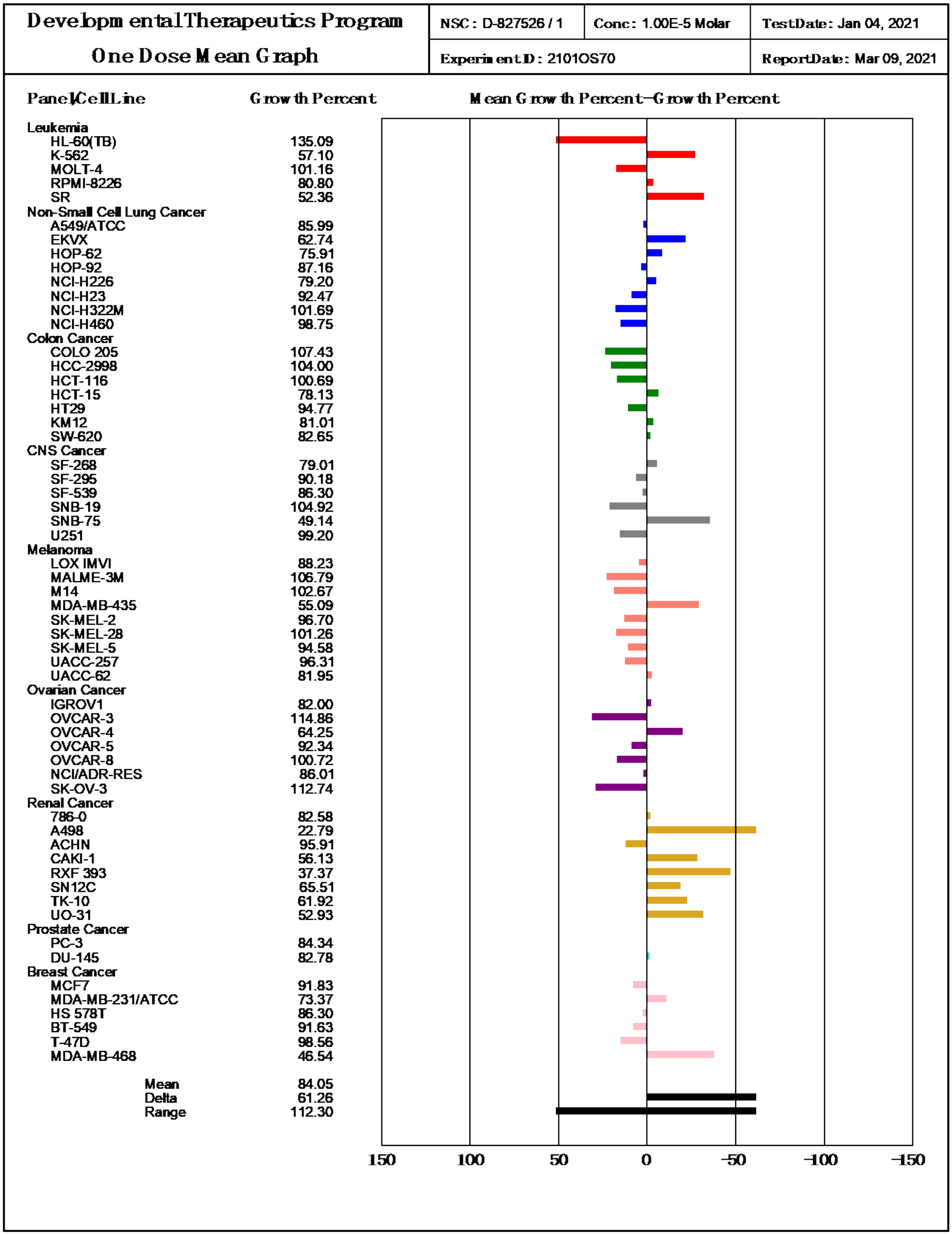

Figure S5. One dose mean growth % of compound **15d** over NCI human cancer cell lines.

Developmental Therapeutics Program	NSC : D-827525 / 1	Conc : 1.00E-5 Molar	Test Date : Jan 04, 2021
One Dose Mean Graph	Experiment ID : 2101OS70		Report Date : Mar 09, 2021

Panel/Cell Line	Growth Percent
**Leukemia**	
HL-60(TB)	116.61
K-562	82.83
MOLT-4	94.88
RPMI-8226	88.71
SR	74.17
**Non-Small Cell Lung Cancer**	
A549/ATCC	83.84
EKVX	68.16
HOP-62	73.48
HOP-92	100.02
NCI-H226	82.16
NCI-H23	98.17
NCI-H322M	100.34
NCI-H460	104.20
**Colon Cancer**	
COLO 205	109.24
HCC-2998	108.24
HCT-116	95.79
HCT-15	90.87
HT29	99.48
KM12	93.71
SW-620	96.41
**CNS Cancer**	
SF-268	91.48
SF-295	86.32
SF-539	87.79
SNB-19	98.93
SNB-75	54.93
U251	101.87
**Melanoma**	
LOX IMVI	82.48
MALME-3M	109.31
M14	102.99
MDA-MB-435	82.33
SK-MEL-2	92.63
SK-MEL-28	101.73
SK-MEL-5	101.68
UACC-257	89.20
UACC-62	74.82
**Ovarian Cancer**	
IGROV1	79.43
OVCAR-3	108.32
OVCAR-4	73.05
OVCAR-5	96.22
OVCAR-8	103.82
NCI/ADR-RES	93.82
SK-OV-3	110.02
**Renal Cancer**	
786-0	73.39
A498	52.60
ACHN	101.22
CAKI-1	47.62
RXF 393	57.01
SN12C	75.02
TK-10	52.96
UO-31	53.61
**Prostate Cancer**	
PC-3	94.82
DU-145	88.71
**Breast Cancer**	
MCF7	94.95
MDA-MB-231/ATCC	80.32
HS 578T	98.13
T-47D	91.98
MDA-MB-468	77.81
Mean	88.15
Delta	40.53
Range	68.99

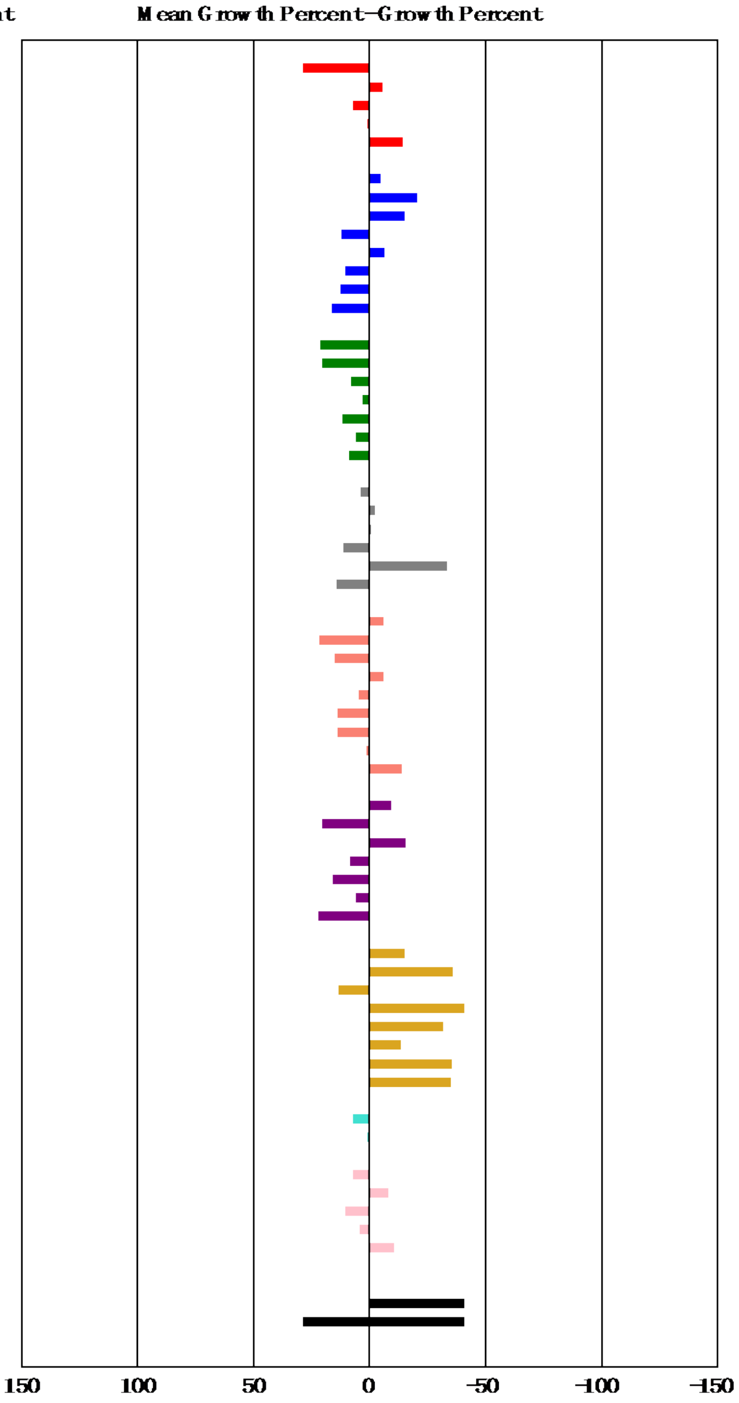

Figure S6. One dose mean growth % of compound **15e** over NCI human cancer cell lines.

Developmental Therapeutics Program	NSC : D-827524 / 1	Conc : 1.00E-5 Molar	Test Date : Jan 04, 2021
One Dose Mean Graph	Experiment ID : 2101OS70		Report Date : Mar 09, 2021

Panel/Cell Line	Growth Percent
**Leukemia**	
HL-60(TB)	123.20
K-562	79.41
MOLT-4	97.69
RPMI-8226	67.40
SR	72.28
**Non-Small Cell Lung Cancer**	
A549/ATCC	74.00
EKVX	60.59
HOP-62	64.36
HOP-92	74.10
NCI-H226	77.19
NCI-H23	75.99
NCI-H322M	85.66
NCI-H460	62.25
**Colon Cancer**	
COLO 205	107.09
HCC-2998	104.33
HCT-116	74.46
HCT-15	100.70
HT29	91.30
KM12	87.60
SW-620	97.11
**CNS Cancer**	
SF-268	87.96
SF-295	64.07
SF-539	90.00
SNB-19	82.55
SNB-75	71.53
U251	90.21
**Melanoma**	
LOX IMVI	79.02
MALME-3M	99.13
M14	90.33
MDA-MB-435	92.28
SK-MEL-2	102.26
SK-MEL-28	99.06
SK-MEL-5	86.83
UACC-257	91.24
UACC-62	69.84
**Ovarian Cancer**	
IGROV1	77.03
OVCAR-3	111.43
OVCAR-4	56.85
OVCAR-5	91.79
OVCAR-8	96.51
NCI/ADR-RES	98.94
SK-OV-3	109.00
**Renal Cancer**	
786-0	75.00
A498	39.28
ACHN	86.68
CAKI-1	44.67
RXF 393	62.60
SN12C	55.15
TK-10	58.73
UO-31	42.31
**Prostate Cancer**	
PC-3	73.80
DU-145	85.21
**Breast Cancer**	
MCF7	103.05
MDA-MB-231/ATCC	71.25
HS 578T	98.24
T-47D	81.06
MDA-MB-468	86.98
Mean	82.08
Delta	42.80
Range	83.92

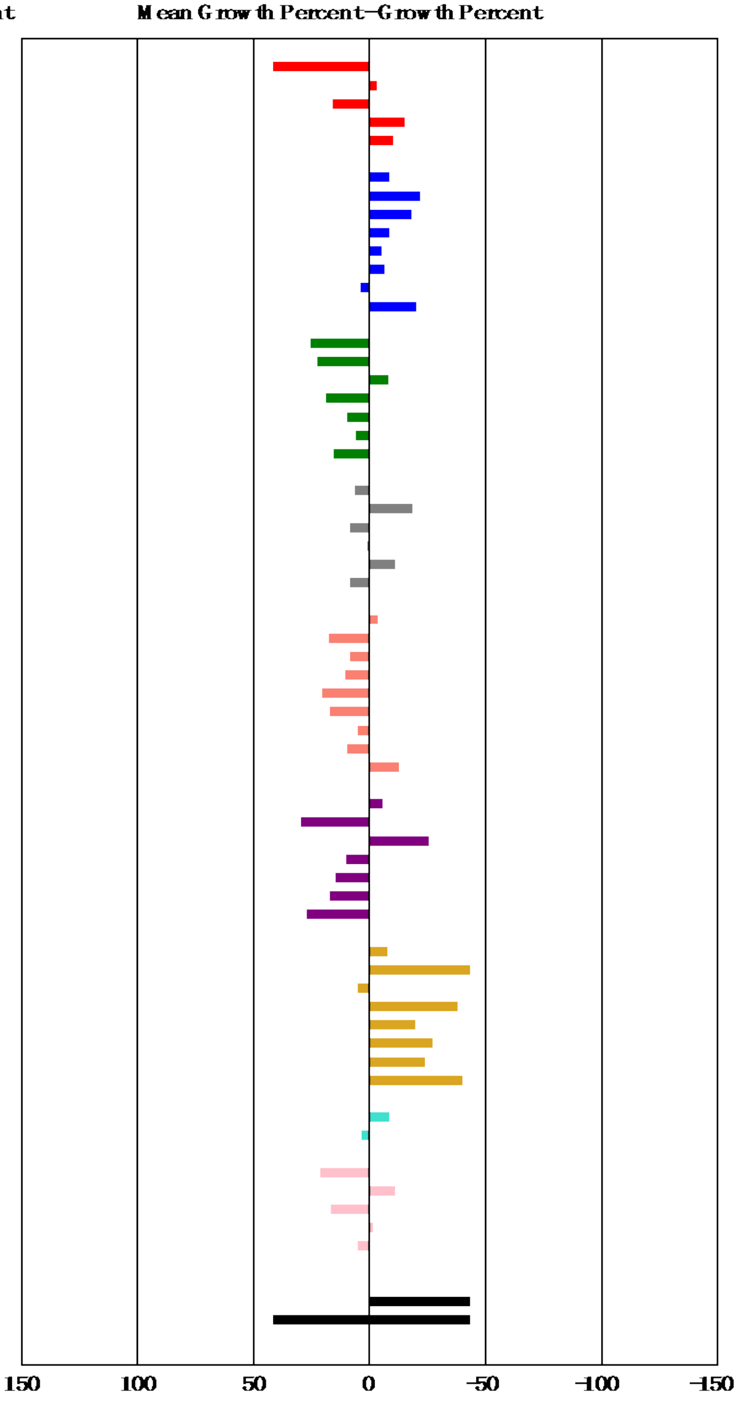

Figure S7. One dose mean growth % of compound **15f** over NCI human cancer cell lines.

Developmental Therapeutics Program	NSC : D-827532 / 1	Conc : 1.00E-5 Molar	Test Date : Jan 04, 2021
One Dose Mean Graph	Experiment ID : 2101OS70		Report Date : Mar 09, 2021

Panel/Cell Line	Growth Percent
**Leukemia**	
HL-60(TB)	149.01
K-562	112.83
MOLT-4	111.19
RPMI-8226	93.08
SR	109.65
**Non-Small Cell Lung Cancer**	
A549/ATCC	91.54
EKVX	69.53
HOP-62	80.88
HOP-92	108.79
NCI-H226	93.94
NCI-H23	94.61
NCI-H322M	105.58
NCI-H460	107.34
**Colon Cancer**	
COLO 205	115.61
HCC-2998	113.69
HCT-116	103.56
HCT-15	116.10
HT29	106.16
KM12	100.78
SW-620	104.17
**CNS Cancer**	
SF-268	88.05
SF-295	98.38
SF-539	98.13
SNB-19	101.20
SNB-75	65.83
U251	107.72
**Melanoma**	
LOX IMVI	105.38
MALME-3M	108.11
M14	110.43
MDA-MB-435	108.92
SK-MEL-2	101.55
SK-MEL-28	111.46
SK-MEL-5	98.87
UACC-257	106.07
UACC-62	94.37
**Ovarian Cancer**	
IGROV1	86.20
OVCAR-3	114.13
OVCAR-4	81.67
OVCAR-5	95.81
OVCAR-8	105.83
NCI/ADR-RES	108.44
SK-OV-3	112.14
**Renal Cancer**	
786-0	88.08
A498	55.39
ACHN	102.08
CAKI-1	69.86
RXF 393	53.24
SN12C	79.07
TK-10	71.82
UO-31	60.99
**Prostate Cancer**	
PC-3	96.16
DU-145	94.02
**Breast Cancer**	
MCF7	100.64
MDA-MB-231/ATCC	89.88
HS 578T	104.20
T-47D	110.12
MDA-MB-468	107.75
Mean	97.90
Delta	44.66
Range	95.77

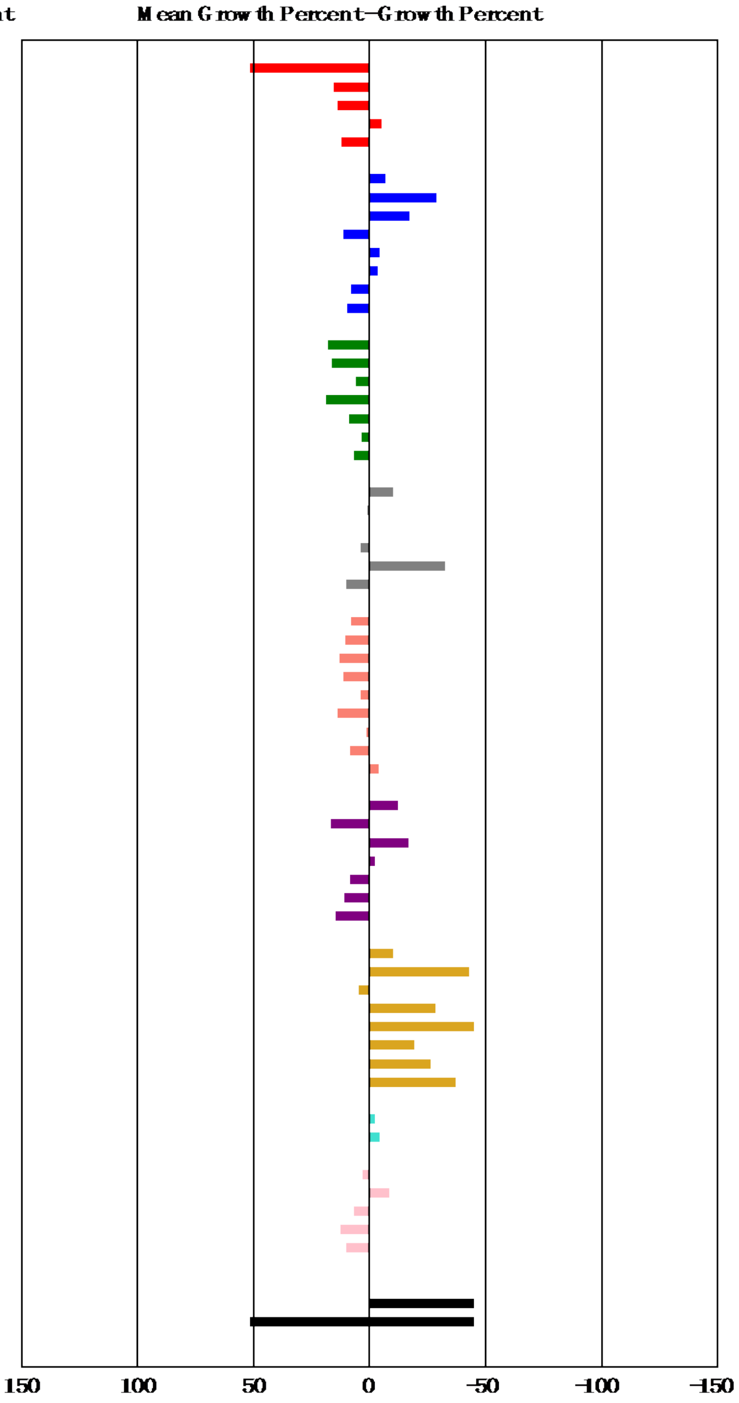

Figure S8. One dose mean growth % of compound **15g** over NCI human cancer cell lines.

Developmental Therapeutics Program	NSC : D-827531 / 1	Conc : 1.00E-5 Molar	Test Date : Jan 04, 2021
One Dose Mean Graph	Experiment ID : 2101OS70		Report Date : Mar 09, 2021

Panel/Cell Line	Growth Percent
**Leukemia**	
HL-60(TB)	130.91
K-562	107.33
MOLT-4	100.78
RPMI-8226	93.67
SR	92.73
**Non-Small Cell Lung Cancer**	
A549/ATCC	86.11
EKVX	75.74
HOP-62	72.72
HOP-92	91.43
NCI-H226	88.27
NCI-H23	97.03
NCI-H322M	106.37
NCI-H460	104.82
**Colon Cancer**	
COLO 205	119.65
HCC-2998	115.16
HCT-116	100.15
HCT-15	112.16
HT29	109.19
KM12	100.08
SW-620	93.73
**CNS Cancer**	
SF-268	84.06
SF-295	97.53
SF-539	96.43
SNB-19	102.38
SNB-75	57.94
U251	105.47
**Melanoma**	
LOX IMVI	98.28
MALME-3M	105.51
M14	106.37
MDA-MB-435	100.17
SK-MEL-2	103.21
SK-MEL-28	111.34
SK-MEL-5	97.49
UACC-257	102.06
UACC-62	89.64
**Ovarian Cancer**	
IGROV1	79.75
OVCAR-3	110.29
OVCAR-4	76.00
OVCAR-5	96.75
OVCAR-8	105.09
NCI/ADR-RES	107.09
SK-OV-3	109.38
**Renal Cancer**	
786-0	87.59
A498	40.80
ACHN	100.25
CAKI-1	66.19
RXF 393	47.77
SN12C	75.90
TK-10	61.89
UO-31	57.01
**Prostate Cancer**	
PC-3	101.18
DU-145	95.62
**Breast Cancer**	
MCF7	98.05
MDA-MB-231/ATCC	86.75
HS 578T	103.70
T-47D	100.92
MDA-MB-468	102.62
Mean	94.15
Delta	53.35
Range	90.11

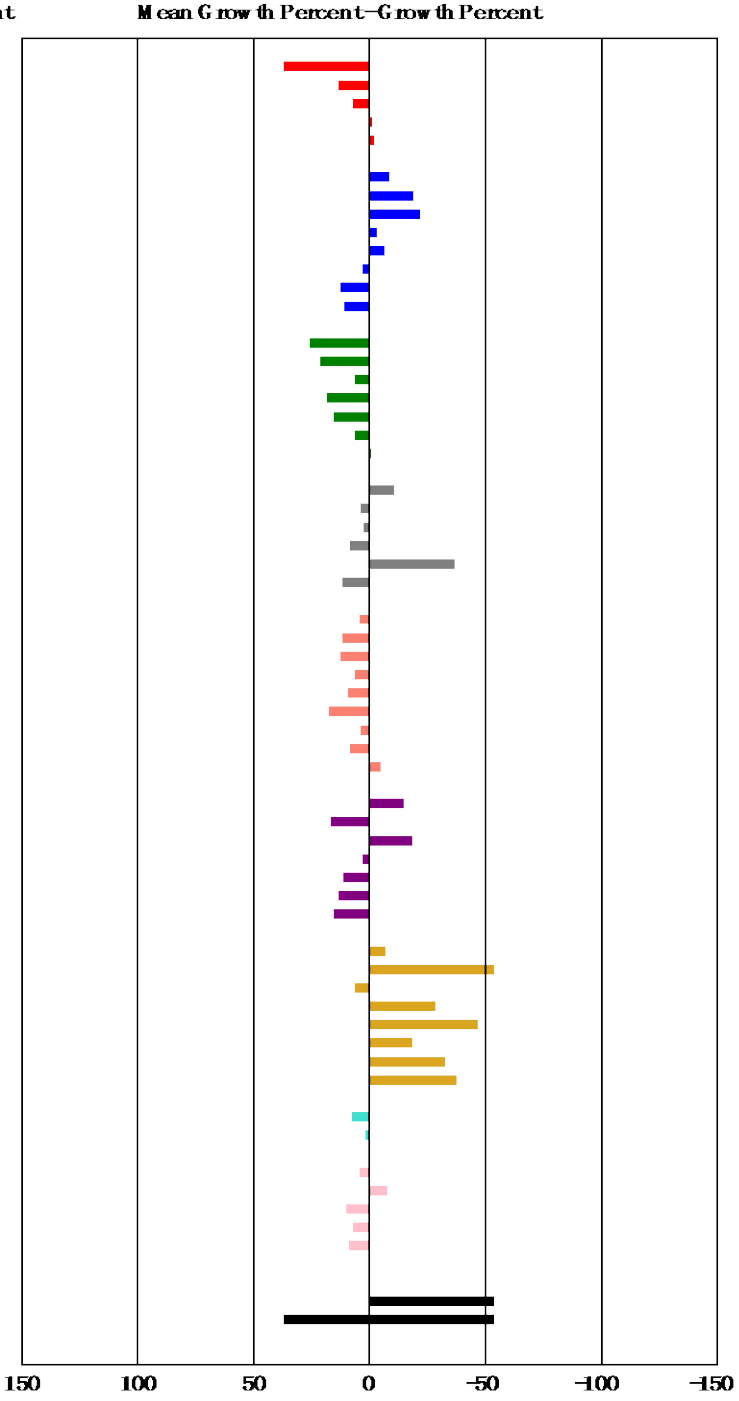

Figure S9. One dose mean growth % of compound **15h** over NCI human cancer cell lines.

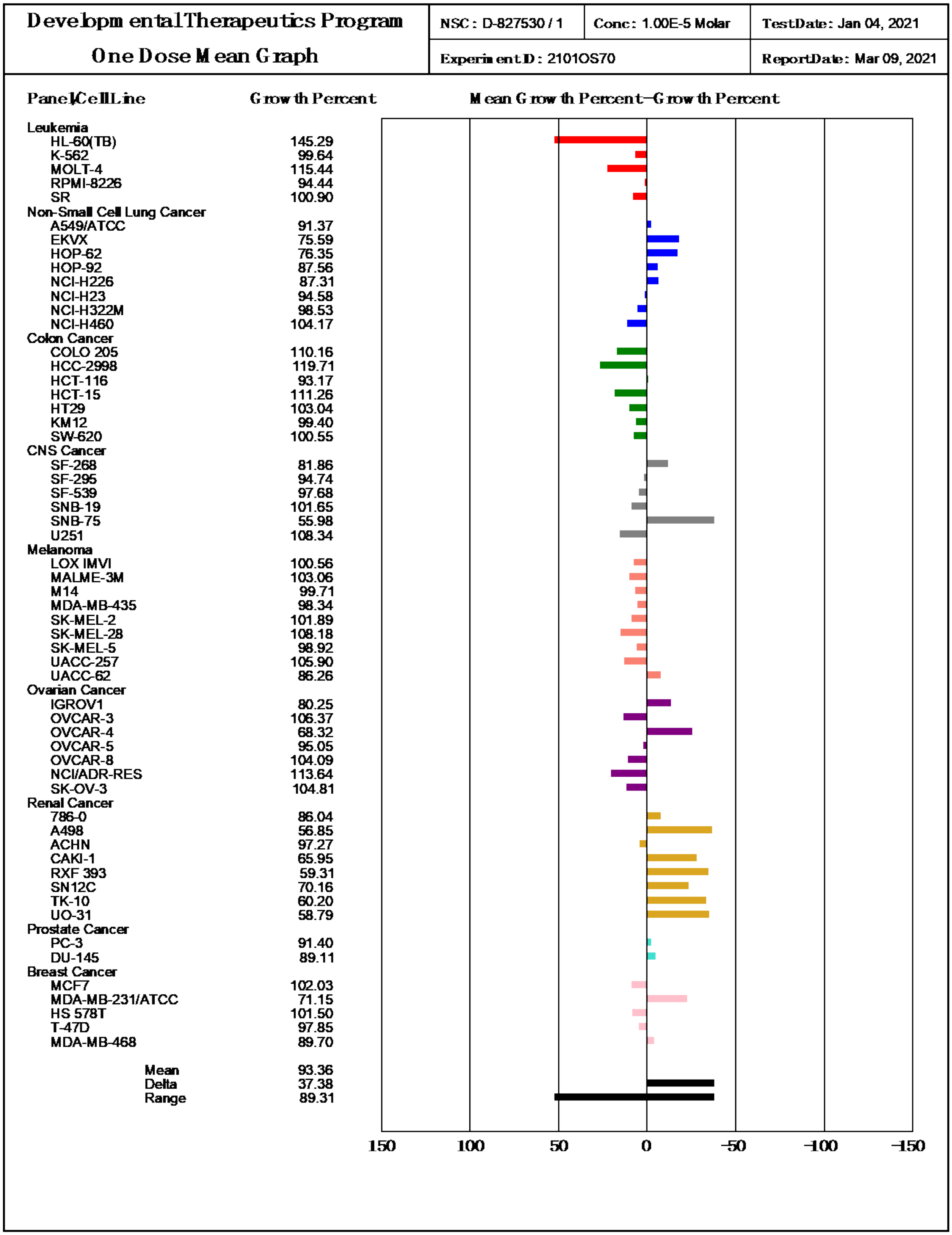

Figure S10. One dose mean growth % of compound **15i** over NCI human cancer cell lines.

Developmental Therapeutics Program	NSC : D-827529 / 1	Conc : 1.00E-5 Molar	Test Date : Jan 04, 2021
One Dose Mean Graph	Experiment ID : 2101OS70		Report Date : Mar 09, 2021

Panel/Cell Line	Growth Percent
**Leukemia**	
HL-60(TB)	110.07
K-562	74.87
MOLT-4	91.56
RPMI-8226	102.73
SR	65.27
**Non-Small Cell Lung Cancer**	
A549/ATCC	86.62
EKVX	73.90
HOP-62	81.30
HOP-92	112.20
NCI-H226	93.39
NCI-H23	99.09
NCI-H322M	103.60
NCI-H460	114.26
**Colon Cancer**	
COLO 205	111.04
HCC-2998	105.80
HCT-116	105.76
HCT-15	98.38
HT29	99.18
KM12	88.83
SW-620	105.71
**CNS Cancer**	
SF-268	92.49
SF-295	88.99
SF-539	100.52
SNB-19	101.50
SNB-75	76.61
U251	109.74
**Melanoma**	
LOX IMVI	95.18
MALME-3M	106.40
M14	108.20
MDA-MB-435	100.78
SK-MEL-2	102.32
SK-MEL-28	104.97
SK-MEL-5	103.39
UACC-257	107.08
UACC-62	89.53
**Ovarian Cancer**	
IGROV1	96.72
OVCAR-3	110.72
OVCAR-4	79.38
OVCAR-5	85.64
OVCAR-8	100.32
NCI/ADR-RES	99.40
SK-OV-3	117.13
**Renal Cancer**	
786-0	86.32
A498	58.74
ACHN	93.23
CAKI-1	40.94
RXF 393	69.83
SN12C	79.50
TK-10	56.14
UO-31	57.80
**Prostate Cancer**	
PC-3	92.39
DU-145	87.94
**Breast Cancer**	
MCF7	98.59
MDA-MB-231/ATCC	89.48
HS 578T	107.65
T-47D	109.76
MDA-MB-468	105.61
Mean	93.59
Delta	52.65
Range	76.19

Mean Growth Percent – Growth Percent

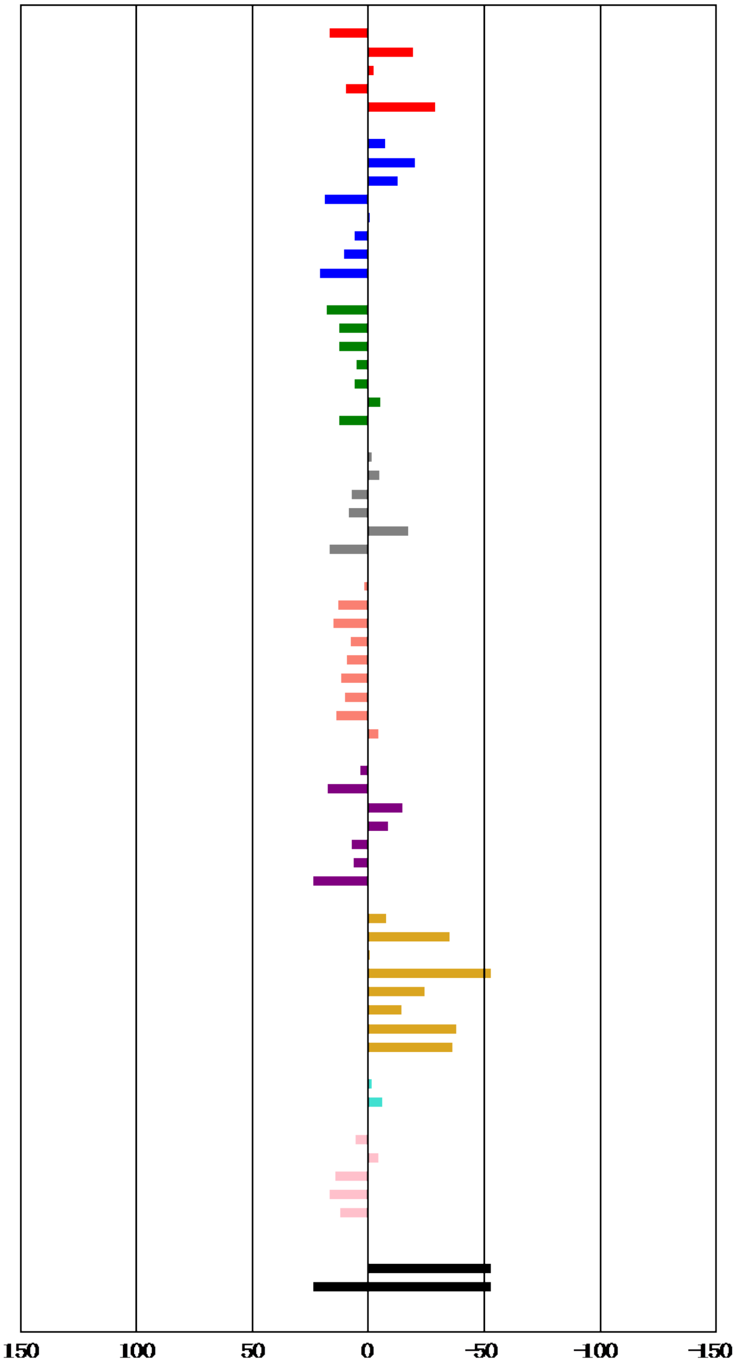

Figure S11. One dose mean growth % of compound **15j** over NCI human cancer cell lines.

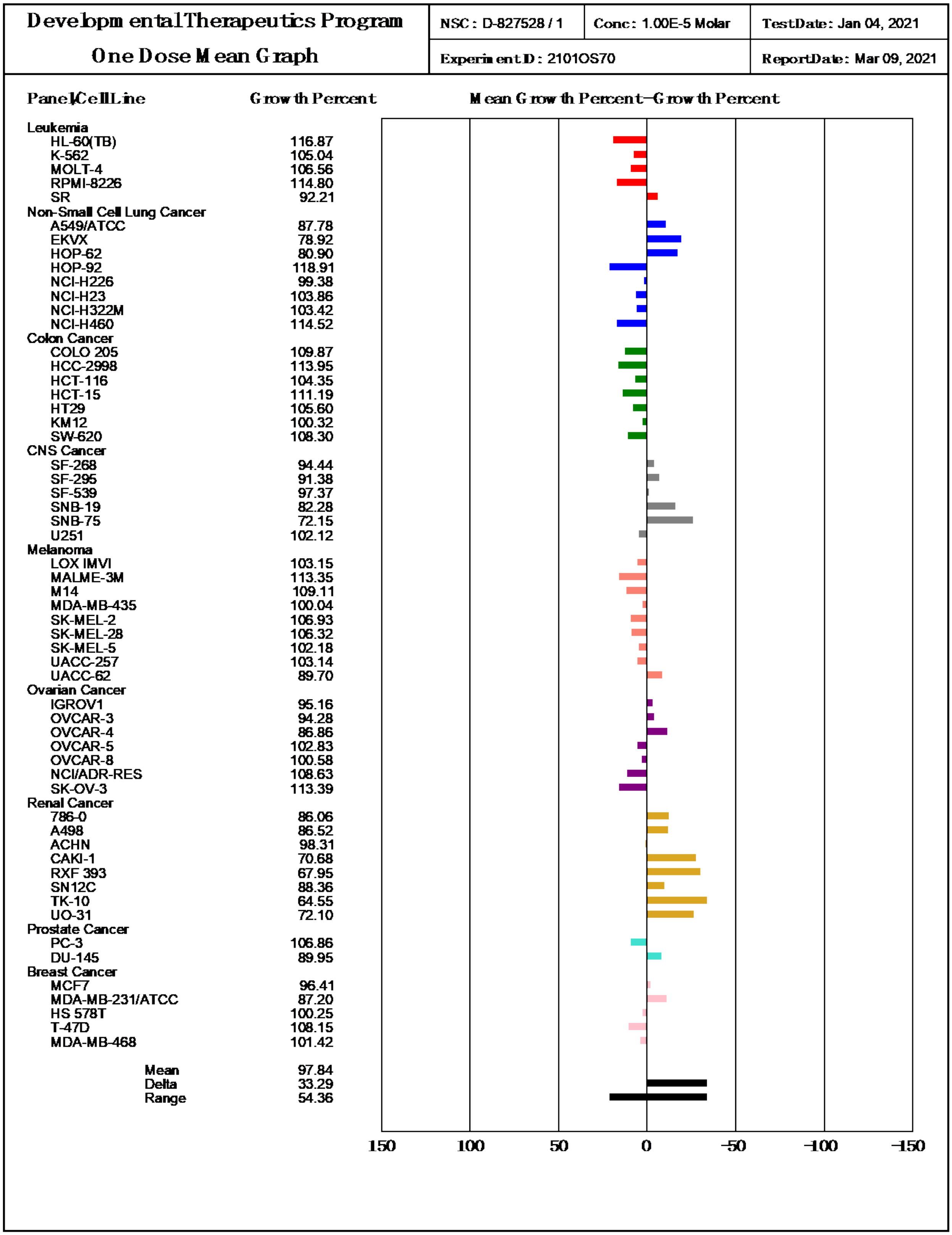

Figure S12. One dose mean growth % of compound **15k** over NCI human cancer cell lines.

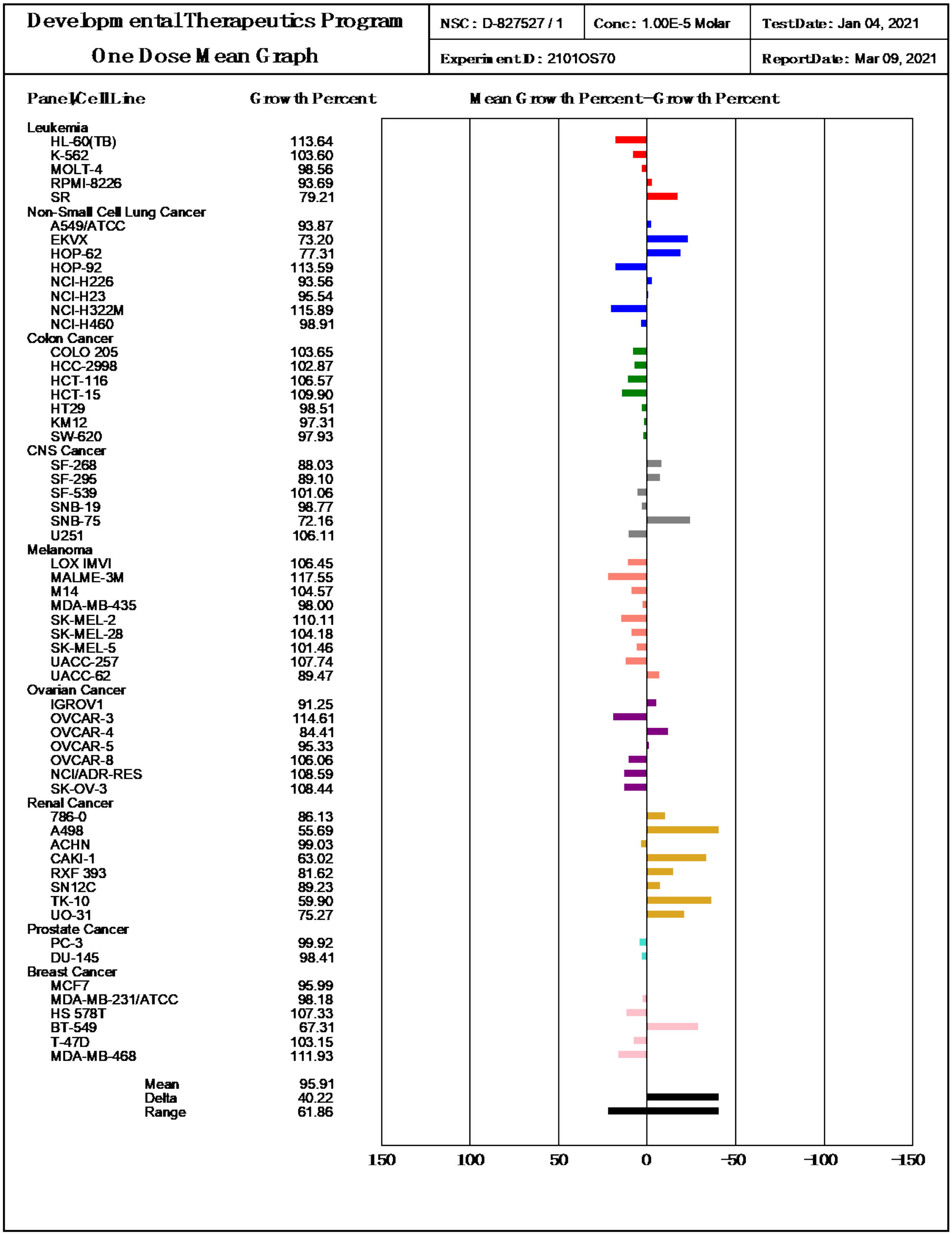

Figure S13. One dose mean growth % of compound **15l** over NCI human cancer cell lines.
